# New Molecules of Diterpene Origin with Inhibitory Properties toward α-Glucosidase

**DOI:** 10.3390/ijms232113535

**Published:** 2022-11-04

**Authors:** Elena Tretyakova, Irina Smirnova, Oxana Kazakova, Ha Thi Thu Nguyen, Alina Shevchenko, Elena Sokolova, Denis Babkov, Alexander Spasov

**Affiliations:** 1 Ufa Institute of Chemistry, Ufa Federal Research Centre, Russian Academy of Sciences, 71 Prospect Oktyabrya, 450054 Ufa, Russia; 2 Institute of Chemistry, Vietnamese Academy of Science and Technology, 18 Hoang Quoc Viet Str., Cau Giay Dist., Hanoi 100000, Vietnam; 3 Scientific Center for Innovative Drugs, Volgograd State Medical University, Novorossiyskaya Str. 39, 400087 Volgograd, Russia

**Keywords:** diabetes mellitus, α-glucosidase, abietane diterpenoids, levopimaric acid, quinopimaric acid, molecular docking, ADMET

## Abstract

The incidence of diabetes mellitus (DM), one of the most common chronic metabolic disorders, has increased dramatically over the past decade and has resulted in higher rates of morbidity and mortality worldwide. The enzyme, α-Glucosidase (α-GLy), is considered a therapeutic target for the treatment of type 2 DM. Herein, we synthesized arylidene, heterocyclic, cyanoetoxy- and propargylated derivatives of quinopimaric acid (levopimaric acid diene adduct with *p*-benzoquinone) **1**–**50** and, first, evaluated their ability to inhibit α-GLy. Among the tested compounds, quinopimaric acid **1**, 2,3-dihydroquinopimaric acid **8** and its amide and heterocyclic derivatives **9**, **30**, **33**, **39**, **44**, with IC_50_ values of 35.57–65.98 μM, emerged as being good inhibitors of α-GLy. Arylidene 1β-hydroxy and 1β,13α-epoxy methyl dihydroquinopimarate derivatives **6**, **7**, **26**–**29**, thiadiazole **32**, 1a,4a-dehydroquinopimaric acid **40** and its indole, nitrile and propargyl hybrids **35**–**38**, **42**, **45**, **48**, and **50** showed excellent inhibitory activities. The most active compounds **38**, **45**, **48**, and **50** displayed IC_50_ values of 0.15 to 0.68 μM, being 1206 to 266 more active than acarbose (IC_50_ of 181.02 μM). Kinetic analysis revealed the most active diterpene indole with an alkyne substituent **45** as a competitive inhibitor with *K*_i_ of 50.45 μM. Molecular modeling supported this finding and suggested that the indole core plays a key role in the binding. Compound **45** also has favorable pharmacokinetic and safety properties, according to the computational ADMET profiling. The results suggested that quinopimaric acid derivatives should be considered as potential candidates for novel alternative therapies in the treatment of type 2 diabetes.

## 1. Introduction

Enzymes responsible for breaking down proteins, carbohydrates and lipids into smaller and more readily absorbable molecules are a key component of the digestive system. The main cause of many metabolic diseases is abnormal changes in the activity of these enzymes. Inhibition of metabolic enzymes, such as α-glucosidase (α-GLy), is one of the accepted approaches in the treatment of diabetes mellitus (DM), which is one of the most common chronic endocrine diseases, along with arterial hypertension and obesity [1]. The number of people with disorders of carbohydrate metabolism and the incidence of DM are constantly growing, which is primarily due to an increase in the number of patients with obesity, as well as in average life expectancy [2]. Type II diabetes mellitus (DM2), accounting for about 90% of all cases of diabetes, is caused by a decrease in insulin sensitivity in target organs, such as the liver, muscle, and adipose tissue, as well as a deficiency in insulin secretion [3,4,5]. Medicinal agents with the ability to stimulate glucose uptake in these tissues can be used to improve insulin resistance and, therefore, to treat DM2 [6]. Today, a vast number of synthetic antidiabetic agents, such as acarbose, miglitol, sulfonylurea, metformin, and thiozolidinedione, are readily available on the market [7,8,9]. However, their effectiveness is limited, due to low bioavailability and unwanted side effects [10,11,12]. Therefore, there is a great need to develop alternative and more active antidiabetic drugs from natural sources.

Abietane diterpenoids are classes of compounds which are mainly found in the conifer family and have long been used to treat a variety of ailments [13]. Their derivatives are characterized by a wide range of biological activities, like anticancer, antiviral, antimicrobial, antileishmanial, antiplasmodial, antifungal, antitumour, cytotoxicity, antiulcer, cardiovascular, antioxidant, anti-inflammatory and antidiabetic activities [14,15,16,17,18,19,20,21]. Abietic and dehydroabietic acids have been reported to decrease the activity of glucose-6-phosphatase and to stimulate glycogen synthase [22]. Carnosic acid derivatives are very effective in treating diabetic complications by improving insulin secretion [23] and glucose homeostasis or by stimulating glucose uptake by increasing peripheral glucose clearance in tissues [24]. Abietic and carnosic acids also significantly activate nuclear receptor peroxisome proliferator-activated receptor PPAR-γ by exerting its beneficial effect on lipid and glucose homeostasis through PPAR-γ-mediated pathways [25,26]. Carnosol stimulates glucose uptake [27], improves diabetes and its complications by the regulation of oxidative stress and inflammatory responses [28] and suppresses forskolin-induced luciferase expression, when monitored by the cAMP/response element, and glucose-6-phosphatase gene promoters [29,30,31]. Tanshinones exhibited potent protein tyrosine phosphatase 1B inhibitory activity [32] as well as increased the activity of insulin on the tyrosine phosphorylation of the insulin receptor in addition to the activation of the kinases Akt, ERK1/2, and GSK3beta and may be very useful for developing new anti-diabetic agents as specific insulin receptor activators [33].

Studies evaluating the antidiabetic properties of abietane diterpenoids in an animal model using rats and mice showed that dehydroabietic acid reduces plasma glucose and insulin levels, as well as plasma and hepatic triglyceride levels, by suppressing the production of monocyte chemoattractant protein-1 and tumor necrosis factor-alpha and increasing that of adiponectin, through decrease in macrophage infiltration into adipose tissues [34]. Carnosol and carnosic acid reduced plasma glucose, total cholesterol, and triglycerides in a diabetic group of rats and suppressed inflammation and lipogenesis in mice administered a high-fat diet, through C-kinase substrate regulation [23,28,35]. Tanshinone analogs also demonstrated a significant decrease in blood glucose level, total cholesterol and triglyceride, free fatty acids, and insulin receptor substrate 1 expression, body weight loss and higher insulin resistance when administered to type 2 diabetic rats, with oral administration, resulting in the activation of AMP-activated protein kinase in aortas from ob/ob or db/db mice [35,36]. Data on systematic studies of the enzymatic activity of abietane diterpenoid derivatives, obtained as a result of various modifications of the native core, in particular, on levopimaric acid derivatives as potential inhibitors of α-GLy, are practically absent in the literature. Therefore, herein, we describe the synthesis of abietane type derivatives with arylidene, heterocyclic, nitrile and acetylene fragments. These derivatives were, then, first evaluated for in-vitro α-GLy inhibition. The mechanism of inhibition and enzyme binding were investigated with kinetic and molecular modeling approaches.

## 2. Results and Discussion

### 2.1. Chemistry

**Figure 1:**
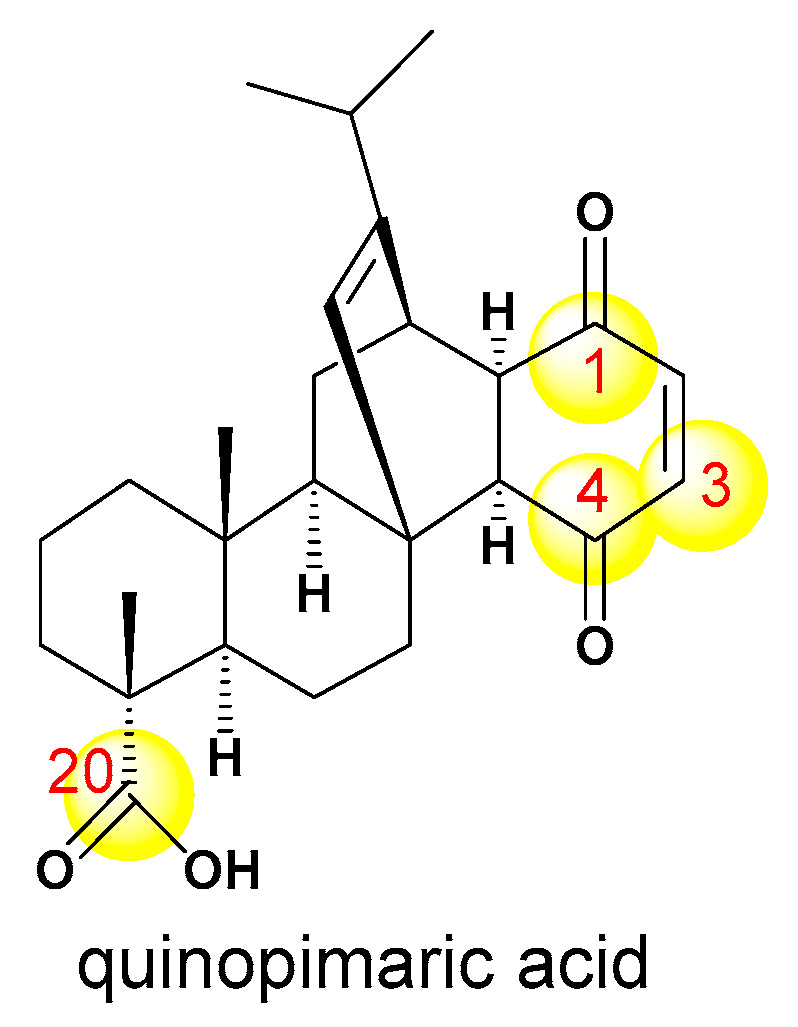
Quinopimaric acid reaction centers for the SAR studies of the current research.

Since the quinopimaric acid structure (the Diels-Alder reaction product of levopimaric acid and *p*-benzoquinone) contains major reaction centers at the C-1, C-3, C-4, and C-20 atoms, we planned to functionalize these positions for better understanding of the structure–activity relationship and to reveal new promising molecules with antidiabetic activity (Figure 1). 

**Figure 2:**
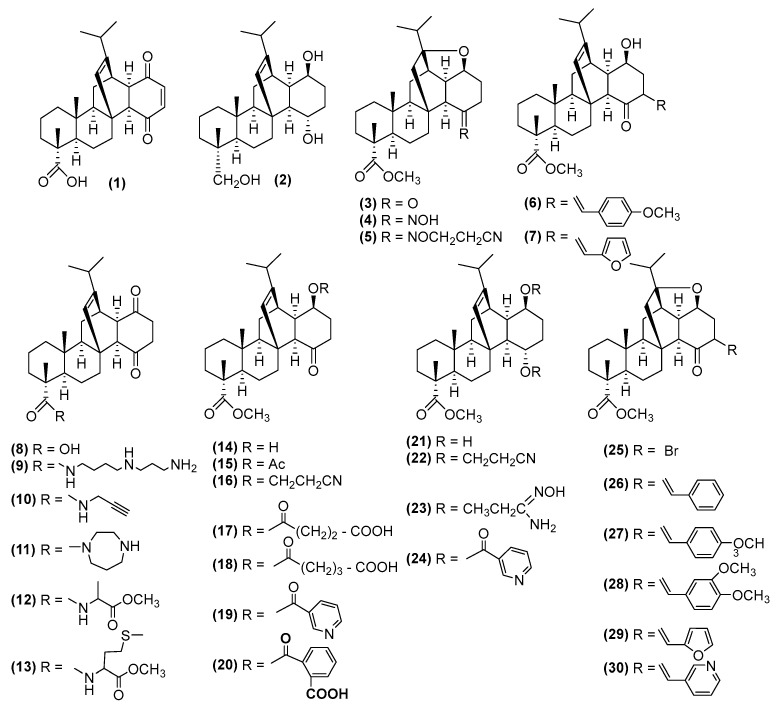
Structures of quinopimaric acid **1** and its analogs **2**–**30**, modified at position C-1, C-3, C-4, and C-20.

Modifications of these sites involved the synthesis of arylidene and heterocyclic derivatives and quinopimaric acid cyanoetoxy- and propargylated analogs. Figure 2 shows the structures of quinopimaric acid **1** and its analogs **2**–**30**, modified at position C-1, C-3, C-4, and C-20.

**Figure 3:**
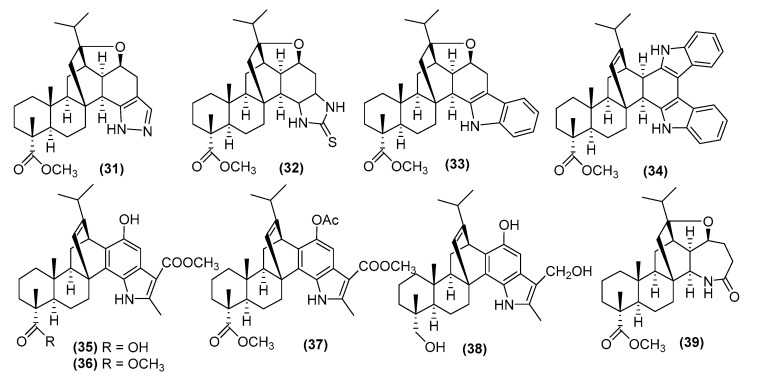
Structures of quinopimaric acid heterocyclic derivatives **31**–**39**.

Figure 3 shows the structures of quinopimaric acid heterocyclic derivatives obtained as a result of interaction with hydrazine hydrate **31**, thiourea **32**, and using the Fischer reaction (indoles **33**, **34**), Nenitzescu reaction (indoles **35**–**38**) and Beckmann rearrangement (lactam **39**).

**Scheme 1:**
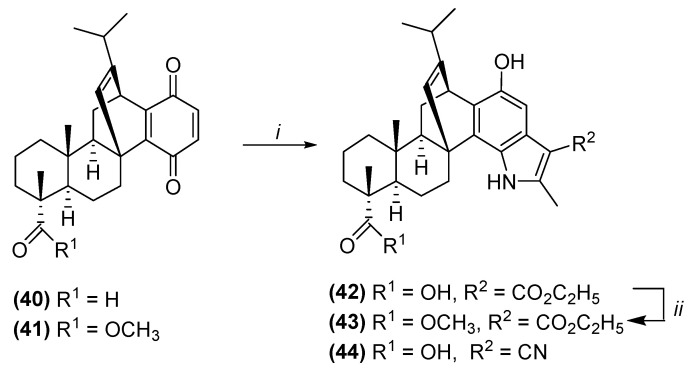
Synthesis of new diterpene indoles **42**–**44** by Nenitzescu reaction. Reagents and conditions: (i) ethyl 3-aminocrotonate for **42**, **43** or 3-aminocrotononitrile for **44**, AcOH, rt, 20 h; (ii) CH_3_I, K_2_CO_3_, acetone, reflux, 2 h.

We planned to use the Nenitzescu reaction [37] for the synthesis of new diterpene indoles. In this reaction, 1a,4a-dehydroquinopimaric acid **40**, easily formed in two steps from quinopimaric acid **1** [38], was used as the quinone component, as well as ethyl 3-aminocrotonate or 3-aminocrotononitrile being were used as the new enamine components. Under the conditions of the Nenitzescu indole synthesis by the reaction of 1a,4a-dehydroquinopimaric acid **40** with the corresponding enamine in glacial AcOH, at room temperature, diterpene indoles **42**, **44** were synthesized in 76 and 69% yields, respectively. Methyl ester of diterpene indole **43** was obtained in quantitative yield by treating compound **42** with methyl iodide during reflux in acetone for 2 h in the presence of potash (Scheme 1), or direct synthesis from 1a,4a-dehydroquinopimaric acid methyl ester **41**, similar to the preparation of compounds **42** and **44**.

**Scheme 2:**
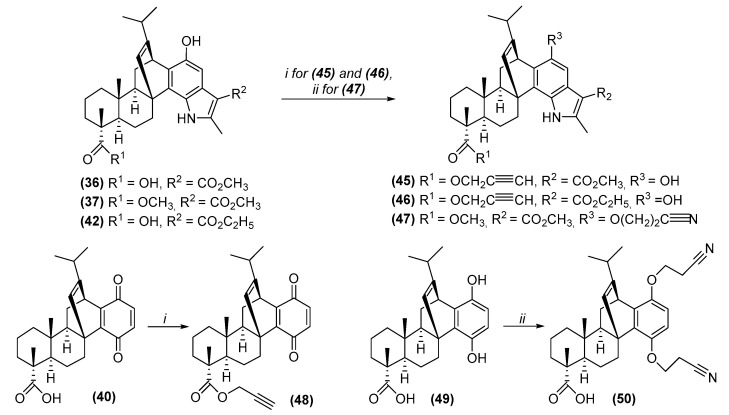
Synthesis of propargyl **45**, **46**, **48** and cyanoethyloxy **47**, **50** quinopimaric acid derivatives. Reagents and conditions: (i) propargyl bromide, DMF, K_2_CO_3_, reflux, 2 h; (ii) acrylonitrile, 1,4-dioxane, KOH, BTAC, rt, 2 h.

Propargyl derivatives **45**, **46**, **48** were obtained in 79–83% yields by the reaction of diterpene indoles **36**, **43** and quinone **40** with propargyl bromide during reflux in dimethylformamide in the presence of K_2_CO_3_. Cyanoethyloxy derivatives **47**, **50** were prepared by adding acrylonitrile in 1,4-dioxane. at room temperature, to the diterpene indole **37** or aromatic derivative **49** in the occurrence of phase transfer catalyst triethylbenzylammonium chloride in combination with an alkali (30% KOH) (Scheme 2).

The structures of the synthesized compounds were confirmed using mass spectrometry, and one- and two-dimensional (COSY, NOESY, ^1^H–^13^C HSQC, ^1^H–^13^C HMBC) NMR spectroscopy. Thus, the signal of the C-2 carbon atom of the aromatic ring in the ^13^C NMR spectra of compound **42**–**44** appeared at δ 99.7-103.3 ppm, and correlated with the signal of the H-2 proton at δ 6.83–7.28 ppm in the ^1^H–^13^C HSQC spectra. The ^1^H NMR spectra showed characteristic signals of methyl group protons at δ 2.51–2.71 (3′-CH_3_), as well as broadened signals of the hydroxyl group and NH group at δ 9.12–9.35 and 12.13 ppm, respectively. The ^1^H NMR spectra of compound **43** contained an additional signal of the protons of the methyl ester group at δ 3.76 ppm, which, in the ^1^H–^13^C HSQC spectrum, correlated with the signal of the C-21 atom at δ 15.5 ppm. In the ^13^C NMR spectra of compound **44**, a carbon signal of the CN-group was observed at δ 117.8 ppm. The ^1^H NMR spectra of propargyl derivatives **45**, **46**, **48** contained the methylene group proton signal in the region δ 4.67–4.82 ppm, while in the ^13^C NMR the triple bond carbon signals appeared at δ 74.4–74.9 and 77.9–79.3 ppm, respectively. The signals of the cyanoethyl methylene groups in the ^1^H NMR spectra of compounds **47**, **50** were observed in the region δ 2.80–2.91 and 4.05–4.30 ppm, and the characteristic carbon signal of the nitrile group in the ^13^C NMR spectra was observed at δ 117.4–117.6 ppm (Figures S1–S18, Supplementary Materials).

### 2.2. Inhibition of Yeast α-Glucosidase

All the synthesized compounds **1**–**50** were tested for their inhibitory potential against yeast α-GLy. Acarbose served as a control drug in this experiment. The IC_50_ values of compounds are provided in Table 1. 

Quinopimaric acid derivatives **2**–**5**, **10**–**25**, **27**, **31**, **34**, **41**, **43**, **46**, **49** showed moderate to poor α-GLy inhibition. Quinopimaric acid **1**, 2,3-dihydroquinopimaric acid **8** and its amide and heterocyclic derivatives **9**, **30**, **33**, **39**, **44**, with an IC_50_ value of 35.24 ± 0.71 Μm—65.98 ± 0.03 μM, emerged as good inhibitors of α-GLy. Arylidene 1β-hydroxy and 1β,13-epoxy methyl dihydroquinopimarate derivatives **6**, **7**, **26**–**29**, thiadiazole **32**, 1a,4a-dehydroquinopimaric acid **40** and its indole, nitrile and propargyl hybrids **35-38**, **42**, **45**, **48**, and **50** showed excellent inhibitory activities. The most active compounds, **38**, **45**, **48**, and **50**, displayed IC_50_ values of 0.15 ± 0.008 μM to 0.68 ± 0.045 μM, being 1206 to 266 more potent than acarbose (IC_50_ of 181.02 ± 3.1 μM).

As shown in Table 1, quinopimaric acid **1** had an activity against α-GLy three times higher than that of acarbose. Its simplest modifications, namely, the reduction of the C2-C3 bond (compound **8**), led to an increase in activity, and the introduction of a double bond into the C1a-C4a position (compound **40**) further enhanced this. Modifications at positions C-1, C-4, C-20 of dihydroquinopimaric acid (compounds **2**–**5**, **9**–**14**, **15**–**24**) were unsuccessful and led to a complete loss of activity. However, the introduction of arylidene substituents into the C-3 position of 1β,13α-epoxy methyldihydroquinopimarate **3** (compounds **26**, **28**–**30**) resulted mainly in active compounds, and the most successful, for this series of compounds, was the presence of a furfural fragment in the molecule. Heterocyclization of dihydroquinopimaric acid (compounds **31**–**34**, **39**) led to compounds with high activity against α-GLy only in the case of thiadiazole **32** and indole **34**.

The use of acid **40** as a starting compound for heterocyclization provided more active compounds. Indole and its derivatives **35**–**38** showed excellent activity, especially alcohol **38**. Inspired by these results, we carried out the synthesis of new indoles using other enamines. The obtained two new indoles **42** and **44** also had very good activity, and it was better for the indole with an ethyl substituent. Modification of the C-20 position by introducing a triple bond into the molecule (compounds **45**, **46**), and the C-1 position with a cyanoethyl fragment (compound **47**) in the case of indole **36**, further enhanced this activity.

Thus, from the studied series of 50 compounds synthesis of indoles based on 1a,4a-dehydroquinopimaric acid **40** proved to be the most successful approach to obtain highly active α-GLy inhibitors. Modification, according to the Nenitzescu reaction, and reduction of carboxyl and methoxycarboxyl groups, with the formation of trihydroxy derivative **38**, propargylation of C-20 positions in acid **40** and indole **36**, as well as cyanoethylation of the aromatic derivative **49**, realized compounds with IC_50_ values < 1 µM.

For compounds **38**, **45**, **48** and **50**, which showed the highest activity against α-GLy, studies of their anti-antioxidant, antimicrobial and cytotoxic activity were carried out (Tables S1–S3, see Supplementary Materials).

### 2.3. The Mechanism of α-Glucosidase Inhibition by Compound ***45***

**Figure 4:**
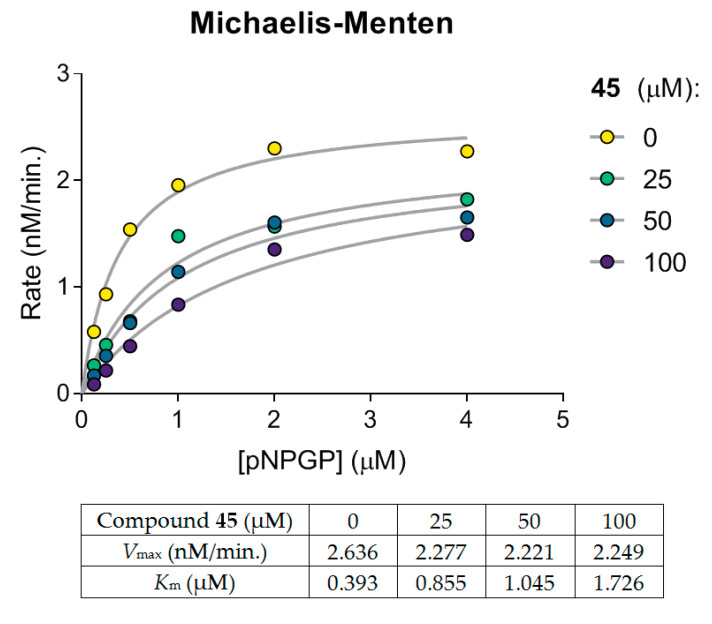
Michaelis—Menten kinetics for compound **45** indicates a competitive inhibition. The experiment was performed in two independent series.

The mechanism of action for the most active diterpene indole with an alkyne substituent **45** was determined in a kinetic experiment using different 4-nitrophenyl β-D-glucopyranoside (pNPG) substrate concentrations. Nonlinear regression of kinetic curves, using the Michaelis–Menten equation (Figure 4), revealed that higher inhibitor concentrations increased *K_m_*, while *V_max_* remained constant, which rendered compound **45** as a competitive inhibitor. The inhibition constant *K_i_* was estimated as 50.45 µM.

The literature data regarding the mechanism of α-glucosidase inhibition for diterpenes is scarce. The majority of reported compounds are non-competitive inhibitors, e.g., ent-atisane-3-oxo-16β,17-acetonide [39], (E)-labda-8(17),12-diene-15,16-dial [40], ent-kaurane derivative of chepraecoxin A [41], and bis-labdanic diterpene were reported as mixed-type inhibitors [42]. Notably, these inhibitors share an alicyclic core. In contrast, diterpene carnosol, comprising aromatic catechol moiety, is a competitive inhibitor [43]. Aromatic rings of carnosol and compound **45** favor π-stacking interaction with phenylalanine, tyrosine, or tryptophan side chains [44], which might result in distinct binding patterns and inhibition kinetics.

### 2.4. Docking Studies for Compound ***45***

**Figure 5:**
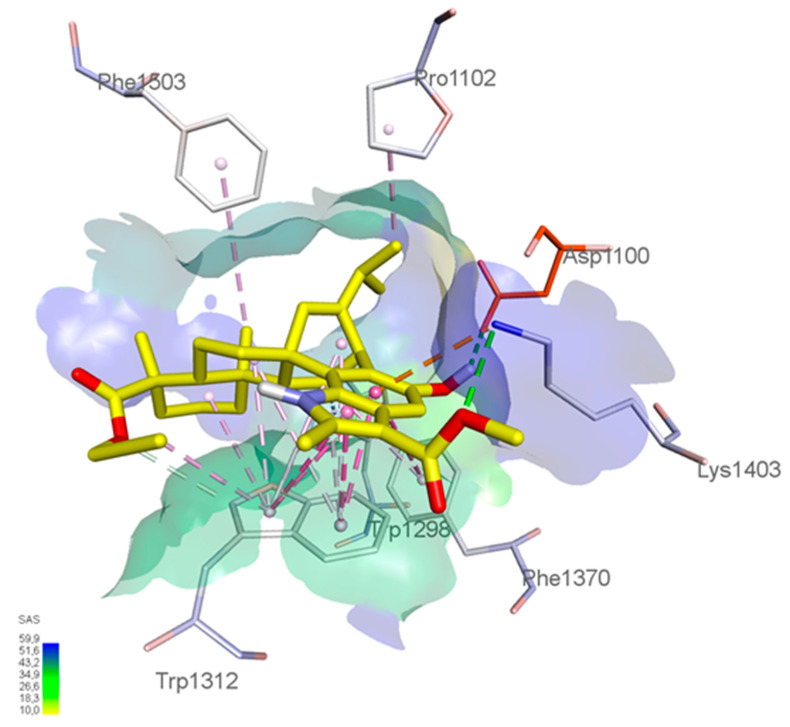
Proposed binding mode of compound **45** to yeast α-glucosidase. The inhibitor is shown in yellow carbons, catalytic Asp1100 is shown in orange carbons. Surface visualized solvent-accessible area. Dashed lines indicate key interactions with enzyme amino acids.

We performed a molecular modeling study to gain insight into the structural basis of interactions between the lead compound **45** and α-GLy enzyme. Since “structure cannot be predicted from kinetics” [45], we avoided preconceived competitive mechanism assumptions and subjected the whole protein surface to a docking procedure (Figure 5). 

Nevertheless, docking proposed that diterpene derivative 45 shared a favorable binding site with acarbose. Moreover, the indole hydroxyl group appeared to form a conventional H-bond with carboxyl of the catalytic Asp1100 residue. The indole core itself contributed to the binding the most. It was anchored by strong π-π parallel stacking with a Trp1312 side chain and T-shaped π-stacking with a Phe1370 side chain. The amino group of Lys1403 formed an H-bond with the ester substituent and charged π-cation interaction with the indole aromatic system. The dodecahydrophenanthrene part of the molecule was also stabilized by Van der Waals forces with multiple lipophilic residues (Pro1102, Trp1298, Trp1312, Phe1503). Both ester moieties of compound 45 pointed towards the solvent-accessible area of the pocket, providing an opportunity for the introduction of polar fragments. This modification might improve water solubility without hampering enzyme binding. To sum up, molecular modeling confirmed the competitive mechanism of action revealed in the kinetic experiment and provided guidance for future structural optimization.

### 2.5. ADMET Profiling of Compound ***45***

We assessed drug-like, pharmacokinetic and toxicological properties of the lead compound **45** using a consensus of predictive services that took into account different computational strategies (Table 2).

Compound **45** fulfilled Lipinski’s rule of 5 (with the exception of molecular weight <500) and Pfizer’s rules. At the same time, GSK and “Golden triangle” rules were violated. Mean water solubility was acceptable. Importantly, there was a good consensus on low intestinal absorption and oral bioavailability, which could avoid systemic exposure to the substance. In the case of entering systemic circulation, indole **45** was anticipated to be bound to plasma proteins, likely due to high lipophilicity. Blood–brain barrier penetration was unlikely. Liver metabolism was to be mediated by cytochrome P450 3A4. Acute oral toxicity was predicted to be sufficiently low to achieve a wide therapeutic window. There were no alerts for toxicity to the liver and heart, nor mutagenicity and carcinogenicity. Hence, compound **45**’s calculated ADMET profile was favorable for the proposed mechanism of action.

## 3. Materials and Methods

### 3.1. General

The spectra were recorded at the Center for the Collective Use “Chemistry” of the UIC UFRC RAS and RCCU “Agidel” of the UFRC RAS. ^1^H and ^13^C-NMR spectra were recorded on a “Bruker AM-500” (Bruker, Billerica, MA, USA, 500 and 125.5 MHz respectively, δ, ppm, Hz) in CDCl_3_, internal standard tetramethylsilane. Melting points were detected on a micro table “Rapido PHMK05“ (Nagema, Dresden, Germany). Optical rotations were measured on a polarimeter “Perkin-Elmer 241 MC” (Perkin Elmer, Waltham, MA, USA) in a tube length of 1 dm. Elemental analysis was performed on a Euro EA-3000 CHNS analyzer (Eurovector, Milan, Italy); the main standard was acetanilide. Thin-layer chromatography analyses were performed on Sorbfil plates (Sorbpolimer, Krasnodar, Russian Federation), using the solvent system chloroform–ethyl acetate, 40:1. Substances were detected by 10% H_2_SO_4_ with subsequent heating to 100–120 °C for 2–3 min. All the reagents and solvents were purchased from standard commercial vendors and were used without any further purification. For the synthesis of quinopimaric acid **1** [38] pine resin Pinus silvestris (containing about 25% levopimaric acid) was used. Compounds **2**, **14**, **15**, **17**–**21**, **24** [50], **3** [51], **4** [52], **5** [53], **6**, **9**, **11**, **26**–**28**, **30** [16], **7** [54], **8** [50], **10** [55], **12**, **13** [56], **16** [15], **22**, **23** [57], **25** [58], **29** [59], **31**, **32**, **34** [60], **33** [14], **35** [61], **36**–**39** [38], **40**, **49** [62] were obtained according to the methods previously described.

### 3.2. Synthesis of Compounds ***42*** and ***44***

A threefold excess of ethyl 3-aminocrotonate (0.387 g, 3 mmol) or 3-aminocrotononitrile (0.246 g, 3 mmol) was added with stirring to a solution of compound **40** (0.408 g, 1 mmol) in glacial AcOH (20 mL). The reaction mixture was stirred at room temperature for 20 h, and then poured into H_2_O. The precipitate was filtered off, washed until neutral, and the residue was air-dried. The reaction product was chromatographed on a silica gel column, eluent CHCl_3_–MeOH, 40: 1.

1-Hydroxy-13-isopropyl-1′-(ethoxycarbonyl)-7,10a,2′-trimethyl-5,6,6b,7,8,9,10,10a, 10b,11,12,13-dodecahydro-12,4b-ethenophenanthro-[2,1-g]indole-7-carboxylic acid (**42**). Yield 0.395 g (76%), mp 131–133°C, [α]_D19_ + 12.2° (c 1.5, CHCl_3_). ^1^H NMR spectrum, δ, ppm (J, Hz): 0.79 (3H, s, 18-CH_3_); 0.86–0.99 (1H, m, 10-CH_2_); 1.18 (3H, d, J = 7.0, 17-CH_3_); 1.22 (3H, d, J = 7.0, 16-CH_3_); 1.29 (3H, s, 19-CH_3_); 1.22–1.69 (10H, m, 6,8,9,10,11-CH_2_, 10b-CH); 1.77–1.80 (1H, m, 6b-CH); 1.98–2.18 (1H, m, 15-CH); 2.45–2.58 (1H, m, 5-CH_2_); 2.67 (3H, s, 3′-CH_3_); 2.82–2.91 (1H, m, 5-CH_2_); 3.45 (3H, s, 6′-CH_3_); 4.27 (1H, s, 12-CH); 4.31-4.35 (2H, m, 5′-CH_2_); 5.68 (1H, s, 14-CH); 7.13 (1H, s, 2-CH); 9.65 (2H, br. s, OH); 9.80 (1H, br. s, NH). ^13^C NMR spectrum, δ, ppm: 14.9 (C-3′); 16.4 (C-19); 16.7 (C-9), 17.2 (C-18); 20.5 (C-17); 20.8 (C-16); 22.0 (C-6); 28.3 (C-11); 32.0 (C-15); 33.5 (C-5); 36.1 (C-12); 36.8 (C-8); 38.2 (C-4b); 38.9 (C-10); 46.5 (C-7); 46.6 (C-10a); 49.8 (C-6b); 50.3 (C-6′); 54.9 (C-10b); 60.2 (C-5′), 102.9 (C-2); 108.0 (C-1′); 124.5 (C-3); 127.5 (C-13); 131.1 (C-1a); 133.6 (C-4a); 143.3 (C-4); 146.8 (C-1); 153.2 (C-14); 162.8 (C-2′); 165.3 (C-4′); 183.1 (C-20). Mass spectrum, m/z (Irel, %): 520 [M+H]^+^ (100). Found, %: C 73.95; H 7.92; N 2.68. C_32_H_41_NO_5_. Calculated, %: C 73.96; H 7.95; N 2.70.

1′-Cyano-1-hydroxy-13-isopropyl-7,10a,2′-trimethyl-5,6,6b,7,8,9,10,10a,10b,11,12,13-dodecahydro-12,4b-ethenophenanthro [2,1-g]indole-7-carboxylic acid (**44**). Yield 0.32 g (69%), mp 127–129°C, [α]_D19_ +25.5° (c 1.0, CHCl_3_). ^1^H NMR spectrum, δ, ppm (J, Hz): 0.78 (3H, s, 18-CH_3_); 0.82–0.95 (1H, m, 10-CH_2_); 1.18 (3H, d, J = 7.0, 17-CH_3_); 1.20 (3H, d, J = 7.0, 16-CH_3_); 1.25 (3H, s, 19-CH_3_); 1.22–1.69 (10H, m, 6,8,9,10,11-CH_2_, 10b-CH); 1.77–1.80 (1H, m, 6b-CH); 1.98–2.18 (1H, m, 15-CH); 2.45–2.58 (1H, m, 5-CH_2_); 2.51 (3H, s, 3′-CH_3_); 2.55–2.85 (1H, m, 5-CH_2_); 4.31 (1H, s, 12-CH); 5.65 (1H, s, 14-CH); 6.55-6.83 (1H, m, 2-CH); 9.35 (3H, br. s, OH, NH). ^13^C NMR spectrum, δ, ppm: 12.8 (C-3′); 16.4 (C-19); 16.7 (C-9), 17.1 (C-18); 20.1 (C-17); 20.3 (C-16); 22.0 (C-6); 28.1 (C-11); 32.0 (C-15); 34.6 (C-5); 36.8 (C-12); 37.6 (C-8); 38.2 (C-4b); 38.8 (C-10); 46.3 (C-7); 46.8 (C-10a); 49.9 (C-6b); 55.2 (C-10b); 83.3 (C-1′); 99.7 (C-2); 117.8 (C-4′); 124.4 (C-3); 126.8 (C-1a); 127.1 (C-14); 130.9 (C-4); 134.4 (C-4a); 144.3 (C-1); 146.4 (C-13); 153.7 (C-2′); 182.5 (C-20). Mass spectrum, m/z (Irel, %): 473 [M+H]^+^ (100). Found, %: C 76.22; H 7.65; N 5.90. C_30_H_36_N_2_O_3_. Calculated, %: C 76.24; H 7.68; N 5.93.

### 3.3. Synthesis of Methyl 1-Hydroxy-13-Isopropyl-1′-(Ethoxycarbonyl)-7,10a,2′-Trimethyl-5,6,6b,7,8,9,10,10a,10b,11,12,13-Dodecahydro-12,4b-Ethenophenanthro-[2,1-g]indole-7-Carboxylate (***43***)

Procedure A. Methyl iodide (2 mL) and potassium carbonate (0.21 g) were added to a solution of **40** (0.408 g, 1 mmol) in acetone (15 mL), and the mixture was heated to reflux for 2 h. The reaction mixture was filtered. The filtrate was evaporated under reduced pressure, and the residue was purified on a silica gel column, eluent hexane–ethyl acetate, 5:1.

Procedure B. A threefold excess of 3-aminocrotononitrile (0.246 g, 3 mmol) was added with stirring to a solution of compound **41** (0.422 g, 1 mmol) in glacial AcOH (20 mL). The reaction mixture was stirred at room temperature for 20 h, and then poured into H_2_O. The precipitate was filtered off, washed until neutral, and the residue was air-dried. The reaction product was chromatographed on a silica gel column, eluent CHCl_3_–MeOH, 40: 1.

Yield 0.45 g (85%), mp 120–122°C, [α]_D19_ +12.9° (c 0.75, CHCl_3_). ^1^H NMR spectrum, δ, ppm (J, Hz): 0.79 (3H, s, 18-CH_3_); 0.86–0.99 (1H, m, 10-CH_2_); 1.18 (3H, d, J = 7.0, 17-CH_3_); 1.22 (3H, d, J = 7.0, 16-CH_3_); 1.29 (3H, s, 19-CH_3_); 1.22–1.69 (10H, m, 6,8,9,10,11-CH_2_, 10b-CH); 1.40–1.43 (3H, s, 6′-CH_3_); 1.77–1.80 (1H, m, 6b-CH); 1.98–2.18 (1H, m, 15-CH); 2.45–2.58 (1H, m, 5-CH_2_); 2.73 (3H, s, 3′-CH_3_); 2.85–3.15 (1H, m, 5-CH_2_); 3.76 (3H, s, 21-CH_3_), 4.27 (1H, s, 12-CH); 4.31–4.39 (2H, m, 5′-CH_2_); 5.71 (1H, s, 14-CH); 7.28 (1H, s, 2-CH); 5.95 (1H, br. s, NH); 12.13 (1H, br. s, OH). ^13^C NMR spectrum, δ, ppm: 14.9 (C-3′); 15.5 (C-21), 16.4 (C-19); 16.8 (C-9), 17.2 (C-18); 20.2 (C-17); 20.5 (C-16); 22.1 (C-6); 28.3 (C-11); 32.2 (C-15); 33.2 (C-5); 36.3 (C-12); 36.9 (C-8); 38.4 (C-4b); 38.9 (C-10); 46.6 (C-7); 47.4 (C-10a); 49.9 (C-6b); 52.0 (C-6′); 54.8 (C-10b); 60.3 (C-5′), 103.3 (C-2); 108.2 (C-1′); 124.7 (C-3); 127.6 (C-13); 130.9 (C-1a); 133.6 (C-4a); 143.6 (C-4); 146.4 (C-1); 153.1 (C-14); 162.9 (C-2′); 165.2 (C-4′); 179.7 (C-20). Mass spectrum, m/z (I_rel_, %): 534 [M] + (100). Found, %: C 74.25; H 8.10; N 2.60. C_33_H_43_NO_5_. Calculated, %: C 74.27; H 8.12; N 2.62.

### 3.4. Synthesis of Compounds ***45***, ***46*** and ***49***

To a solution containing 1 mmol of compound **36**, **37** or **40** in 5 mL of dimethylformamide, 1.2 mmol (0.09 mL) of propargyl bromide and 2.2 mmol (0.30 g) of K_2_CO_3_ were added. The reaction mixture was stirred for 18 h and evaporated at room temperature. The residue was diluted with CHCl_3_, washed with 5% HCl and water, dried over CaCl_2_, and evaporated in a vacuum. The residue was purified by column chromatography, eluent hexane: ethyl acetate, 5:1

Methyl 7-propynyl 1-hydroxy-13-isopropyl-7,10a,2′-trimethyl- 5,6,6b,7,8,9,10,10a,10b,11,12,13-dodecahydro-12,4b-ethenophenanthro [2,1-g]indole-7,1′-dicarboxylate (**45**). Yield 0.39 g (72%), mp 110–112°C, [α]_D20_ + 33.4° (c 0.10, CHCl_3_). ^1^H NMR spectrum, δ, ppm (J, Hz): 0.81 (3H, s, 18-CH_3_); 0.86–0.96 (1H, m, 10-CH_2_); 1.02 (3H, d, J = 7.0, 17-CH_3_); 1.05 (3H, d, J = 7.0, 16-CH_3_); 1.21 (3H, s, 19-CH_3_); 1.31–1.69 (10H, m, 6,8,9,10,11-CH_2_, 10b-CH); 1.77–1.80 (1H, m, 6b-CH); 1.98–2.18 (1H, m, 15-CH); 2.41–2.48 (1H, m, 5-CH_2_); 2.50 (1H, br s., 8′-CH); 2.71 (3H, s, 3′-CH_3_); 2.99–3.00 (1H, m, 5-CH_2_); 3.91 (3H, s, 5′-CH_3_), 4.28 (1H, s, 12-CH); 4.69–4.82 (2H, m, 6′-CH_2_); 5.71 (1H, s, 14-CH); 7.28 (1H, s, 2-CH); 6.00 (1H, br. s, OH); 12.01 (1H, br. s, NH). ^13^C NMR spectrum, δ, ppm: 14.9 (C-3′); 16.8 (C-19); 17.1 (C-9), 18.2 (C-18); 20.2 (C-17); 20.5 (C-16); 22.0 (C-6); 28.3 (C-11); 32.2 (C-15); 33.2 (C-5); 36.3 (C-12); 36.9 (C-8); 38.3 (C-4b); 38.8 (C-10); 46.6 (C-7); 47.4 (C-10a); 49.8 (C-6b); 51.4 (C-6′); 52.0 (C-5′); 54.8 (C-10b); 74.4 (C-8′); 78.1 (C-7′); 103.3 (C-2); 108.0 (C-1′); 124.5 (C-3); 127.5 (C-13); 130.9 (C-1a); 133.6 (C-4a); 143.5 (C-4); 146.5 (C-1); 153.2 (C-14); 162.9 (C-2′); 165.6 (C-4′); 178.2 (C-20). Mass spectrum, m/z (I_rel_, %): 543 [M]+ (100). Found, %: C 75.15; H 7.61; N 2.60. C_34_H_41_NO_5_. Calculated, %: C 75.11; H 7.60; N 2.58.

Ethyl 7-propynyl 1-hydroxy-13-isopropyl-7,10a,2′-trimethyl- 5,6,6b,7,8,9,10,10a,10b, 11,12,13-dodecahydro-12,4b-ethenophenanthro [2,1-g]indole-7,1′-dicarboxylate (**46**). Yield 0.42 g (75%), mp 98–100°C, [α]_D20_ +2.9° (c 0.15, CHCl_3_). ^1^H NMR spectrum, δ, ppm (J, Hz): 0.72 (3H, s, 18-CH_3_); 0.80–0.96 (1H, m, 10-CH_2_); 1.02 (3H, d, J = 7.0, 17-CH_3_); 1.05 (3H, d, J = 7.0, 16-CH_3_); 1.21 (3H, s, 19-CH_3_); 1.31–1.69 (10H, m, 6,8,9,10,11-CH_2_,10b-CH); 1.77–1.80 (1H, m, 6b-CH); 1.98–2.18 (1H, m, 15-CH); 2.41–2.48 (1H, m, 9′-CH); 2.65–2.70 (1H, m, 5-CH_2_); 2.73 (3H, s, 3′-CH_3_); 2.95–3.05 (1H, m, 5-CH_2_); 3.73 (3H, s, 6′-CH_3_), 3.95 (1H, s, 12-CH); 4.35-4.42 (2H, m, 5′-CH_2_); 4.75 (2H, br.s., 6′-CH_2_); 5.70 (1H, s, 14-CH); 7.49 (1H, s, 2-CH); 9.19 (1H, br. s, OH); 12.01 (1H, br. s, NH). ^13^C NMR spectrum, δ, ppm: 14.1 (C-3′); 14.2 (C-19); 14.5 (C-9), 15.9 (C-18); 16.5 (C-17); 16.9 (C-16); 20.2 (C-6); 21.4 (C-11); 22.7 (C-15); 28.3 (C-5); 29.7 (C-12); 32.2 (C-8); 33.2 (C-4b); 36.2 (C-10); 36.9 (C-7); 38.8 (C-10a); 49.9 (C-6b); 51.9 (C-7′); 54.9 (C-6′); 57.1 (C-10b); 60.0 (C-5′), 74.9 (C-9′); 79.3 (C-8′); 101.8 (C-2); 108.6 (C-1′); 124.5 (C-3); 127.6 (C-13); 132.8 (C-1a); 133.8 (C-4a); 144.0 (C-4); 148.6 (C-1); 153.2 (C-14); 162.8 (C-2′); 164.8 (C-4′); 179.5 (C-20). Mass spectrum, m/z (I_rel_, %): 558 [M]+ (100). Found, %: C 75.30; H 7.75; N 2.55. C_35_H_43_NO_5_. Calculated, %: C 75.37; H 7.77; N 2.51.

Propynyl 13-isopropyl-7,10a-dimethyl-1,4-di oxo-4,5,6,6a,7,8,9,10,10a,10b,11,12- dodecahydro-1H-4b,12-ethenochrysene-7-carboxylate (**48**). Yield 0.33 g (75%), mp 85-89°C, [α]_D20_ +26.9° (c 0.75, CHCl_3_). ^1^H NMR spectrum, δ, ppm (J, Hz): 0.69 (3H, s, 18-CH_3_); 0.86–0.96 (1H, m, 10-CH_2_); 1.02 (3H, d, J = 7.0, 17-CH_3_); 1.05 (3H, d, J = 7.0, 16-CH_3_); 1.21 (3H, s, 19-CH_3_); 1.31–1.69 (10H, m, 6,8,9,10,11-CH_2_, 10b-CH); 1.77–1.80 (1H, m, 6b-CH); 1.98–2.18 (1H, m, 15-CH); 2.41–2.48 (1H, m, 5-CH_2_); 2.50 (1H, br. s, 3′-CH); 2.85–2.89 (1H, m, 5-CH_2_); 4.13 (1H, s, 12-CH); 4.67 (2H, br. s, 1′-CH_2_); 5.63 (1H, s, 14-CH); 6.45-6.56 (2H, m, 2-CH, 3-CH). ^13^C NMR spectrum, δ, ppm: 16.4 (C-19); 16.8 (C-9), 17.1 (C-18); 20.2 (C-17); 20.6 (C-16); 21.7 (C-6); 27.1 (C-11); 31.5 (C-15); 31.9 (C-5); 36.2 (C-12); 36.4 (C-8); 38.6 (C-4b); 39.3 (C-10); 47.1 (C-7); 49.1 (C-10a); 49.4 (C-6b); 52.1 (C-1′); 54.8 (C-10b); 74.6 (C-3′); 77.9 (C-2′); 127.3 (C-13); 133.6 (C-2); 137.5 (C-3); 150.6 (C-4a); 151.1 (C-14); 152.8 (C-1a); 177.7 (C-20); 184.0 (C-1); 185.3 (C-2). Mass spectrum, m/z (I_rel_, %): 446 [M]+ (100). Found, %: C 78.05; H 7.65. C_29_H_34_O_4_. Calculated, %: C 78.00; H 7.67.

### 3.5. Synthesis of Compounds ***47*** and ***50***

A mixture of 1 mmol of the compounds **42** or **49**, 20 mmol (1.3 mL) of acrylonitrile and 0.5 mL of 30% KOH per one hydroxyl groups, 0.5 mmol (0.11 g) of BTEAC, in 20 mL of dioxane was stirred for 2 h at room temperature. The mixture was poured into a mixture of ice with HCl, the precipitate was filtered off, washed with water until neutral pH, air dried, and extracted with methylene chloride (3 × 80 mL) with heating, the solution was filtered. The filtrate was evaporated under reduced pressure, and the residue was purified on a silica gel column, eluent hexane–ethyl acetate, 10:1.

Methyl 1-(8′-cyanoethoxy)-13-isopropyl-1′-(ethoxycarbonyl)-7,10a,2′-trimethyl -5,6,6b,7,8,9,10,10a,10b,11,12,13-dodecahydro-12,4b-ethenophenanthro [2,1-g]indole-7-carboxylate (**47**). Yield 0.39 g (68%), mp 127–129 °C, [α]_D20_ +77.9° (c 0.10, CHCl_3_). ^1^H NMR spectrum, δ, ppm (J, Hz): 0.79 (3H, s, 18-CH_3_); 0.86–0.99 (1H, m, 10-CH_2_); 1.04 (3H, d, J = 7.0, 17-CH_3_); 1.07 (3H, d, J = 7.0, 16-CH_3_); 1.24 (3H, s, 19-CH_3_); 1.27–1.69 (10H, m, 6,8,9,10,11-CH_2_, 10b-CH); 1.77–1.80 (1H, m, 6b-CH); 1.98–2.18 (1H, m, 15-CH); 2.45–2.58 (1H, m, 5-CH_2_); 2.76 (3H, s, 3′-CH_3_); 2.89–2.91 (2H, m, 7′-CH_2_); 2.95–3.15 (1H, m, 5-CH_2_); 3.75 (3H, s, 21-CH_3_), 3.93 (3H, s, 5′-CH_3_), 4.28-4.30 (2H, m, 6′-CH_2_); 4.37 (1H, s, 12-CH); 5.72 (1H, s, 14-CH); 7.28 (1H, s, 2-CH); 12.10 (1H, br. s, NH). ^13^C NMR spectrum, δ, ppm: 14.9 (C-3′); 15.5 (C-21), 16.9 (C-19); 16.8 (C-9), 17.2 (C-18); 20.2 (C-17); 20.5 (C-16); 22.1 (C-6); 28.3 (C-11); 32.2 (C-15); 33.2 (C-5); 36.3 (C-12); 36.9 (C-8); 38.4 (C-4b); 38.9 (C-10); 46.7 (C-7); 47.4 (C-10a); 49.9 (C-6b); 51.3 (C-7′); 52.0 (C-5′); 54.9 (C-10b); 63.7 (C-6′), 100.9 (C-2); 108.3 (C-1′); 117.4 (C-8′); 124.6 (C-3); 127.6 (C-13); 133.4 (C-1a); 134.1 (C-4a); 144.0 (C-4); 148.4 (C-1); 153.2 (C-14); 163.0 (C-2′); 165.1 (C-4′); 179.6 (C-20). Mass spectrum, m/z (I_rel_, %): 572 [M]+ (100). Found, %: C 73.45; H 7.75; N 4.91. C_35_H_44_N_2_O_5_. Calculated, %: C 73.40; H 7.74; N 4.89.

1,4-Bis(2′-cyanoethoxy)-13-isopropyl-7,10a-dimethyl-6,6a,7,8,9,10,10a,10b,11,12-decahydro-5H-4b,12-ethenochrysene-7-carboxylic acid (**50**). Yield 0.41 g (80%), mp 157–159 °C, [α]_D20_ + 63.6° (c 0.1, CHCl_3_). ^1^H NMR spectrum, δ, ppm (J, Hz): 0.79 (3H, s, 18-CH_3_); 0.86–0.99 (1H, m, 10-CH_2_); 1.08 (3H, d, J = 7.0, 17-CH_3_); 1.10 (3H, d, J = 7.0, 16-CH_3_); 1.29 (3H, s, 19-CH_3_); 1.22–1.69 (10H, m, 6,8,9,10,11-CH_2_, 10b-CH); 1.77–1.80 (1H, m, 6b-CH); 1.98–2.18 (1H, m, 15-CH); 2.45–2.58 (1H, m, 5-CH_2_); 2.80–2.82 (4H, m, 2′-CH_2_, 2″-CH_2_); 2.95–3.00 (1H, m, 5-CH_2_); 4.05-4.13 (4H, m, 1′-CH_2_, 1″-CH_2_); 4.24 (1H, s, 12-CH); 5.71 (1H, s, 14-CH); 6.38-6.47 (2H, m, 2-CH, 3-CH); 9.12 (1H, br. s, OH). ^13^C NMR spectrum, δ, ppm: 16.4 (C-19); 16.8 (C-9), 17.2 (C-18); 19.9 (C-2′, C-2″); 20.2 (C-17); 20.5 (C-16); 22.1 (C-6); 28.3 (C-11); 32.2 (C-15); 33.2 (C-5); 36.3 (C-12); 36.9 (C-8); 38.4 (C-4b); 38.9 (C-10); 46.6 (C-7); 47.4 (C-10a); 49.9 (C-6b); 54.8 (C-10b); 64.4 (C-1′, C-1″); 111.2 (C-1); 114.4 (C-2); 117.6 (C-3′, C-3″), 128.6 (C-14); 135.2 (C-4a); 138.4 (C-1a); 145.1 (C-4); 146.0 (C-1); 151.7 (C-13); 185.6 (C-20). Mass spectrum, m/z (I_rel_, %): 517 [M]+H (100). Found, %: C 74.35; H 7.81; N 5.40. C_32_H_40_N_2_O_4_. Calculated, %: C 74.39; H 7.80; N 5.42.

Data of the study α-Gly inhibition in vitro, kinetic, docking studies and ADMET profiling of compound **45**. as well as studies of anti-antioxidant, antimicrobial and cytotoxic activities, can be found in the Supplementary Material (Section S1).

## 4. Conclusions

The screening of a series of 50 semisynthetic derivatives of levopimaric acid revealed that, in contrast to the majority of previously reported diterpene α-GLy inhibitors, a lead diterpene indole with an alkyne substituent **45** was identified as a competitive inhibitor. As a consequence, one might hope for better translatability to animal and clinical settings, since the active site of yeast α-GLy and intestinal mammalian maltase–glucoamylase are conserved, while allosteric sites are likely to be different. In addition, compound **45** is anticipated to have low intestinal absorption that benefits high concentration of the drug in the target area and helps to avoid systemic exposure. Additional experiments are warranted to confirm antihyperglycemic properties of compound **45** in vivo. In the event of the efficacy and safety being confirmed, novel glucosidase inhibitors open a promising venue to antidiabetic agents able not only to ameliorate postprandial hyperglycemia, but also reduce secretory load on pancreatic beta-cells.

## Figures and Tables

**Table 1:** α-Glucosidase inhibitory potential of the synthesized compounds **1–50**.

Compound	IC_50_ ± SE (µM)
**1**	59.59 ± 0.18
**2**	>255
**3**	>255
**4**	>255
**5**	>255
**6**	1.63 ± 0.006
**7**	2.50 ± 0.011
**8**	35.57 ± 0.92
**9**	35.24 ± 0.71
**10**	>255
**11**	>255
**12**	>255
**13**	>255
**14**	>255
**15**	>255
**16**	>255
**17**	>255
**18**	>255
**19**	>255
**20**	>255
**21**	>255
**22**	>255
**23**	>255
**24**	>255
**25**	>255
**26**	13.08 ± 0.01
**27**	>255
**28**	12.73 ± 0.21
**29**	1.63 ± 0.041
**30**	38.80 ± 0.33
**31**	>255
**32**	9.66 ± 0.77
**33**	65.98 ± 0.03
**34**	>255
**35**	7.95 ± 0.20
**36**	8.94 ± 0.96
**37**	7.28 ± 0.40
**38**	0.39 ± 0.03
**39**	44.77 ± 0.96
**40**	7.088 ± 0.12
**41**	>255
**42**	2.52 ± 0.34
**43**	>255
**44**	68.22 ± 0.03
**45**	0.15 ± 0.008
**46**	>255
**47**	4.95 ± 0.25
**48**	0.68 ± 0.045
**49**	>255
**50**	0.23 ± 0.01
Acarbose (reference drug)	181.02 ± 3.1

**Table 2:** ADMET properties predicted for compound **45**.

Property	ADMETlab [46]	ADMETlab 2.0 [47]	SwissADME [48]	ProTox-II [49]	Consensus Value
**Physicochemical**					
Water solubility (μg/mL)	1.94	2.57	0.02		1.51
LogP	6.21	4.93	6.30	6.69	5.81
**Absorption**					
Human intestinal absorption	Yes	No	Low		No
Human oral bioavailability	No	No			No
Caco-2 permeability	Yes	No			−
P-glycoprotein substrate	No	No	No		No
P-glycoprotein inhibitor	Yes	Yes			Yes
**Distribution**					
Plasma protein binding (%)	87.09	99.83			93.46
BBB permeability	No	No	No		No
**Metabolism**					
CYP1A2 inhibitor	No	No	No		No
CYP2C19 inhibitor	Yes	Yes	No		Yes
CYP2C9 inhibitor	Yes	Yes	Yes		Yes
CYP2D6 inhibitor	No	Yes	No		No
CYP2D6 substrate	No	No			No
CYP3A4 inhibitor	Yes	Yes	No		Yes
CYP3A4 substrate	Yes	Yes			Yes
**Excretion**					
Total Clearance (mL/min/kg)	1.77	4.03			2.9
T_1/2_ (h)	2.13	0.15			1.14
**Toxicity**					
AMES toxicity	No	No		No	No
hERG inhibitor	Yes	No			−
Rat acute oral LD_50_ (mg/kg)	121.4			520	320.7
Hepatotoxicity	Yes	No		No	No
Skin Sensitisation	No	No			No
Carcinogenicity		No		No	No

## Data Availability

Not applicable.

## Supplementary Materials

The following supporting information can be downloaded at: https://www.mdpi.com/article/10.3390/ijms232113535/s1. Ref [63,64,65,66,67,68,69,70,71] are cited in Supplementary Materials.
