# Supplementary material for: New Molecules of Diterpene Origin with Inhibitory Properties toward α-Glucosidase
Source: Int J Mol Sci. 2022 Nov 4;23(21):13535. doi: 10.3390/ijms232113535 (PMC9655717; doi:10.3390/ijms232113535)
Supplement: Supplementary file 1 [file ijms-23-13535-s001.zip › ijms-1995431-supplementary.pdf]

Supplementary Materials

Inhibitory Properties of Diterpene Quinopimaric Acid Derivatives toward α-Glucosidase

Elena Tretyakova [1], Irina Smirnova [1], Oxana Kazakova [1,*], Ha Thi Thu Nguyen [2], Alina Shevchenko [3], Elena Sokolova [3], Denis Babkov [3] and Alexander Spasov [3]

[1] Ufa Institute of Chemistry, Ufa Federal Research Centre, Russian Academy of Sciences, 71 Prospect Oktyabrya, 450054 Ufa, Russia

[2] Institute of Chemistry, Vietnamese Academy of Science and Technology, 18 Hoang Quoc Viet Str., Cau Giay Dist., Hanoi 100000, Vietnam

[3] Scientific Center for Innovative Drugs, Volgograd State Medical University, Novorossiyskaya Str. 39, 400087 Volgograd, Russia

* Correspondence: obf@anrb.ru; Tel.: +7-347-235-6066

Abstract: The incidence of diabetes mellitus (DM), one of the most common chronic metabolic disorders, has increased dramatically over the past decade and has resulted in higher rates of morbidity and mortality worldwide. α-Glucosidase (α-GLy) is considered a therapeutic target for the treatment of type 2 DM. Herein, we have synthesized arylidene, heterocyclic, cyanoetoxy- and propargylated derivatives of the quinopimaric acid (levopimaric acid diene adduct with *p*-benzoquinone) **1-50** and first evaluated for their ability to inhibit α-GLy. Among tested compounds quinopimaric acid **1**, 2,3-dihydroquinopimaric acid **8** and its amide and heterocyclic derivatives **9**, **30**, **33**, **39**, **44** with the IC_{50} values of 35.57 – 65.98 μM emerged as a good inhibitor of α-GLy. Arylidene 1β-hydroxy and 1β,13α-epoxy methyl dihydroquinopimarate derivatives **6**, **7**, **26-29**, thiadiazole **32**, 1a,4a-dehydroquinopimaric acid **40** and its indole, nitrile and propargyl hybrids **35-38**, **42**, **45**, **48**, and **50** showed the excellent inhibitory activities. The most active compounds **38**, **45**, **48**, and **50** displayed IC_{50} value of 0.15 to 0.68 μM being 1206 to 266 more active than acarbose (IC_{50} of 181.02 μM). Kinetic analysis revealed the most active diterpene indole with an alkyne substituent **45** as a competitive inhibitor with K_i of 50.45 μM. Molecular modeling supported this finding and suggested that the indole core plays a key role in the binding. Compound **45** also has favorable pharmacokinetic and safety properties according to the computational ADMET profiling. The results suggested that quinopimaric acid derivatives be considered as potential candidates for novel alternative therapies in the treatment of type 2 diabetes.

Keywords: diabetes mellitus; α-glucosidase; abietane diterpenoids; levopimaric acid; quinopimaric acid; molecular docking; ADMET

Table S1. The result of antioxidant tests (DPPH) of compounds **38**, **45**, **48** and **50**.

Compound	IC_{50} values (μg/ml)
**38**	227.76±4.9
**45**	>256
**48**	177.45±3.5
**50**	105.50±2.2
**Quercetin**	8.01±0.15

Free radical scavenging ability of compounds **38**, **45**, **48** and **50** was tested by DPPH radical scavenging assay as described by Ha [1]. The hydrogen atom donating ability of the plant extractives was determined by the decolorization of methanol solution of 2,2-diphenyl-1-picrylhydrazyl (DPPH). DPPH produces purple color in methanol solution and fades to shades of yellow color in the presence of antioxidants. Table S1 showed the free radical scavenging activity of compounds **38**, **45**, **48** and **50** and standard Quercetin. The results indicated that, derivatives **38, 48** and **50** were weak activity with IC_{50} value of 227.76 ± 4.9, 177.45 ± 3.5 and 105.50 ± 2.2 μg/mL, respectively. Compound **45** was inactive at the studied concentrations with IC_{50} > 256 μg/mL. The IC_{50} of standard was 8.01 ± 0.15 μg/mL.

Table S2. The result of antimicrobial tests of compounds **38**, **45**, **48** and **50**.

Compound	Values (µg/ml)	Gram (+) strains			Gram (-) strains			Fungi
		S. aureus	*B. subtilis*	*L. fermentum*	*S. enterica*	*E. coli*	*P. aeruginosa*	*C. albican*
**38**	IC_{50}	34.29±1.75	102.4±7.21	>256	>256	>256	>256	>256
	MIC	>256	256	>256	>256	>256	>256	>256
**50**	IC_{50}	10.98±0.83	11.26±0.77	115.51±6.93	>256	>256	>256	>256
	MIC	64	64	>256	>256	>256	>256	>256
**48**	IC_{50}	8.29±0.58	45.04±2.18	43.54±2.52	>256	>256	>256	>256
	MIC	64	256	256	>256	>256	>256	>256
**45**	IC_{50}	>256	>256	>256	>256	>256	>256	>256
	MIC	>256	>256	>256	>256	>256	>256	>256

The antimicrobial activity was determined by broth microdilution method as reported by [2]. Broth dilution testing is one of the common methods used for determination of the MICs and IC_{50}. The compounds are examined for visible bacterial growth as evidenced by turbidity. The results are given in the Table S2. Out of all derivatives, **50** was found to be the most active compound among other derivatives since it significantly inhibited growth activity two gram-positive bacterial *S. aureus, B. subtilis* and produced the MIC value of 64 µg/ml and the IC_{50} value was 10.98 ± 0.83 and 11.26 ± 0.77 µg/ml, respectively. Derivative **48** showed the highest activity against *S. aureus* with MIC value of 64 µg/ml and IC_{50} value was 8.29±0.58 µg/ml. It possessed the weak activity on *L. fermentum* and *B. subtilis* with MIC value was 256 µg/ml and IC_{50} values of 43.54 ± 2.52 µg/ml and 45.04 ± 2.18 µg/ml, respectively. Compound **38** weakly inhibited the growth of *B. subtilis* with MIC value of 256 µg/ml and IC_{50} was 102.4±7.21 µg/ml. These derivatives showed no activity for gram- negative bacteria.

Table S3. The result of cytotoxicity tests of compounds **38**, **45**, **48** and **50**.

Compound	IC_{50} values (μg/ml)			
	KB	Hep-G2	Lu-1	MCF-7
**38**	39.55±1.0	50.78±1.2	58.15±1.8	60.44±1.6
**50**	57.3±1.2	110.22±2.6	69.81±1.5	157.71±4.0
**48**	30.49±0.7	46.0±0.8	10.15±0.18	39.25±0.78
**45**	42.18±0.84	47.17±1.0	16.0±0.36	30.22±0.8
**Ellipticine**	0.31±0.05	0.35±0.05	0.45±0.05	0.5±0.05

The cytotoxic activity of compounds was evaluated against four cancer cell lines KB, Hep-G2, Lu-1 and MCF-7, using colorimetric MTT assay [3]. As resulted in Table S3, derivative **48** has generated the good IC_{50} value of 10.15 ± 0.18 μg/ml repellent the growth of Lu-1 cell line, and exhibited weak activity against three remaining cells with IC_{50} values ranging from 30.49 ± 0.7 to 46.0 ± 0.8 μg/ml. Similarly, compound **45** was also found to inhibit Lu-1 cell line with the moderate IC_{50} values of 16.0 ± 0.36 μg/ml, but suppress KB, Hep-G2 and MCF7 cell lines with the weak IC_{50} values range of 30.22 ± 0.8 - 47.17 ± 1.0 μg/ml. Derivative **38** exhibited the weak cytotoxic activity against four cancer cell lines with the IC_{50} values ranging from 39.55±1.0 to 60.44±1.6 μg/ml. Compound **50** had the least activity with IC_{50} values range of 57.3±1.2-157.71±4.0 μg/ml as compared with those of ellipticine (the IC_{50} values range of 0.31±0.05- 0.5±0.05 μg/ml).

S1. Materials and Methods

S1.1. *In vitro α*-Glucosidase inhibition study of compounds 1-50

The antidiabetic role of synthesized compounds was established by using *α* glucosidase inhibition assay. α-Glucosidase enzyme inhibition assay was carried out in a 96-well microplate. The method's principle based on the hydrolyzed reaction of 4-nitrophenyl-α-D-glucopiranoside with α-glucosidase enzyme into yellow 4-nitrophenol:

$$\textbf{p-Nitrophenyl }\boldsymbol{\alpha}\textbf{-D-Glucoside} \xrightarrow{\text{a-Glucosidase}} \boldsymbol{\alpha}\textbf{-D-Glucose + p- Nitrophenol}$$

α-Glucosidase inhibitory activity assay was performed following the modified method of Pistia Brueggeman and Hollingsworth with slight modification [4, 5]. Quinopimaric acid **1** and its derivatives **2-50** were dissolved in DMSO - EtOH (1 **:** 1) solution to final concentrations of 256, 64, 16, 4 and 1 g/mL. In a 96-well plate, a reaction mixture containing 20 μL of phosphate buffer (100 mM; pH 6.8), 20 μL of *α*-glucosidase (0.3 U/mL, Sigma G0660) (this concentration was determined by a series of *α*-glucosidase kinetic experiments. This enzyme concentration showed the highest coefficient of linearity between the enzyme concentration and the absorbance of yellow color mixture after reaction which was approximately 5-fold higher than the back ground and at least 10-fold smaller than the positive control) and 20 μL of compounds **1-50** of varying concentrations was pre-incubated for 10 min at 37°C. Then 20 μL of 2.5M *p*-nitrophenyl α-D-glucopyranoside (Sigma N1377) was added to the mixture as a substrate. After further incubation at 37°C for 30 min, the reaction was stopped by adding 80 μL of 0.1 M sodium carbonate. The enzyme, inhibitor (one of compounds **1-50**) and substrate solution were prepared using the phosphate buffer 10 μM; pH 6.8. Acarbose was used as a positive control and water as negative control. The yellow color produced was quantitated by colorimetric analysis and reading the absorbance at 410 nm. Each experiment was performed in triplicates, along with appropriate blanks. The solvent DMSO was use as negative control to evaluate its effect on *α*-glucosidase activity. Buffer was used instead of compounds **1-50** in positive control and instead of enzyme or substrate in negative control. Back ground was determined by volume of buffer as reaction mixture.

The % inhibition has been obtained using the formula:

% inhibition = {Absorbance (control) –Absorbance (sample)}/Absorbance (control)

IC_{50} value is defined as the concentration of samples inhibiting 50% of *α*-glucosidase activity under the stated assay conditions.

S1.2. Kinetic study

The kinetic assay was performed according to the method using a series of compound **45** dilutions prepared in 100 mM phosphate buffer (pH 6.8). In a 96-well plate, a reaction mixture containing 10 μL of compound **45** of varying concentrations, 40

μL of 100 mM phosphate buffer (pH 6.8), 25 μL of *α*-glucosidase (0.4 U/mL, Sigma G0660) were pre-incubated for 10 min at 37 °C. DMSO was added to the respective control wells to a 0.64% final concentration. The reaction was initiated with the addition of 25 μL of *p*-nitrophenyl-β-D-glucopyranoside (pNPG, Sigma N1377) solution in a range of final concentrations. The amount of released *p*-nitrophenol from pNPG was immediately measured at 410 nm with Infinite M200 PRO (Tecan, Austria). Conversion of optical density to nM p-nitrophenol was performed with a standard curve. Reaction rates were determined from the linear parts of kinetic curves and fitted to the Michaelis-Menten equation. The assay was performed in triplicate wells and two independent series.

S1.3. Molecular docking

Ligands were prepared with MarvinSketch 18.8.0 (ChemAxon Ltd.) [6]. Ligands were hydrogenized and conformations with minimal potential energy were obtained using the MMFF94 force field. The homology model of *S. cerevisiae α*-glucosidase was obtained with SWISS-MODEL as described previously [7]. Protein and ligand structures followed standard preparation procedure using AutoDocTools 1.5.6. We used AutoDock Vina 1.1.211 to perform all docking runs [8]. The grid box with 70×80×80 Å dimensions was centered on the enzyme molecule to include the entire 3D space around the protein. The top-score binding poses were used in subsequent analysis. Protein-ligand interactions were analyzed with Discovery Studio Visualizer 17.2.0.16349 (Dassault Systemes Biovia Corp.) [9].

S1.4. *In vitro* antioxidant activity (DPPH) study of compounds 38, 45, 48 and 50

DPPH (0.1 mM) was diluted in methanol. 200 μL of this solution was added to 1.3 μL of various concentrations of compounds in DMSO (256.0, 64.0, 16.0, and 4.0 μg/mL). The mixture was performed by a 96-well plate at 25°C in 30 min. Then, absorbance was determined by Epoch™ Microplate *Spectrophotometer* (at 517 nm). The percentage of DPPH quenching activity was computed by the following formula:

$$\text{Inhibitory percentage SC (\%)} = [(A_o - A_1)/A_o] \times 100.$$

where A_O was defined as the absorbance of control reaction, and A_1 represented for the absorbance in the presence of test or standard sample. Each experiment was repeated three times. Quercetin was used as a reference compound. The IC_{50} value, also known as the concentration of tested samples that induced half maximal response has been calculated from linear regression of the serial SC values versus the concentrations by using Table Curve 2Dv4.

S1.5. *In vitro* antimicrobial activity study of compounds 38, 45, 48 and 50

The *Gram-positive bacteria*: *Bacillus subtilis* (ATCC 6633), *Lactobacillus fermentum* (N4), *Staphylococcus aureus* (ATCC 13709); Gram -negative bacteria: *Escherichia coli* (ATCC 25922), *Pseudomonas aeruginosa* (ATCC 15442), *Salmonella enterica*; Yeast: *Candida albicans* (ATCC 10231) were obtained from American Type Culture Collection. The bacterial

culture medium (MHB - Mueller Hinton Broth, TSB - Tryptic Soy Broth, SDB - Sabourand 2% dextrose broth, were purchased from Merck.

Compounds were dissolved in DMSO 100% and deionized water to perform the serial concentrations 256.0, 64.0, 16.0, 4.0 and 1μg/mL. Microbacteria were kept remaining at –80 0 C. Before assay, they were activated by culture medium and, adjusted the concentration to $5x10^5$ CFU/mL. 10 μL of each concentration of each test sample combined with 190 μL of microbacterial solution in 96-well plate which were further incubated at 37^oC within 16-24h. The experiment was triplicated. Positive controls were wells with a bacterial suspension in growth medium, and culture medium without bacteria as a negative control. Ampicillin was used as a reference compound. The results were described as the absorption at 590 nm and calculated by Rawdata software.

The minimum inhibitory concentration (MIC) was defined as the lowest concentration of samples inhibiting the visible growth of the tested microorganisms. The half maximal inhibitory concentration (IC_{50}) was the concentration of samples inhibiting 50% the visible growth of the tested microorganisms. MIC, IC_{50} values are typically expressed as molar concentration.

S1.6. *In vitro* cytotoxicity study of compounds 38, 45, 48 and 50

MTT assay was used to determine the cytotoxic activity of compounds with human cancer cell lines acquired from the American Type Culture Collection (ATCC): epidermoid carcinoma (KB, ATCC number CCL-17), hepatocellular carcinoma (HepG2, ATCC number HB-8065), lung adenocarcinoma LU-1 (ATCC number HTB - 57™), breast adenocarcinoma MCF-7 (ATCC number HTB - 22™. Cells were cultured in medium DMEM supplemented with 10% Fetal bovine serum (FBS), 1% Penicillin and Streptomycin and 1% L-glutamine, under a humidified atmosphere of 5% CO_2 at 37°C.

Compounds were dissolved in DMSO at 40 mg/mL and a series of dilutions for each compound was prepared to final concentrations of 256, 64, 16, 4 and 1 μg/mL. Cells were separated with trypsin and seeded in each well with $3 x10^4$ cells per ml and were treated with sample different concentrations on 96-well plates. Untreated cells represented the controls. After 72h of treatment, an MTT solution (10 μl, 5 mg/mL) of phosphate buffer was added to each well for 4 h until intracellular purple formazan crystals are visible. Remove MTT and add DMSO solution (100 μL). The optical density of the solution was determined by Epoch™ Microplate *Spectrophotometer* at 540 nm. The inhibition ratio was calculated based on the optical densities from the three replicate tests.

IC_{50} ≤ 20 μg/ml (with crude extract) and IC50 ≤ 4 μg/ml or 10 μM (with compound) are evaluated to have cytotoxic activity.

Figure S1. ¹H NMR and ¹³C NMR spectra of compound **42** ($CDCl_3$).

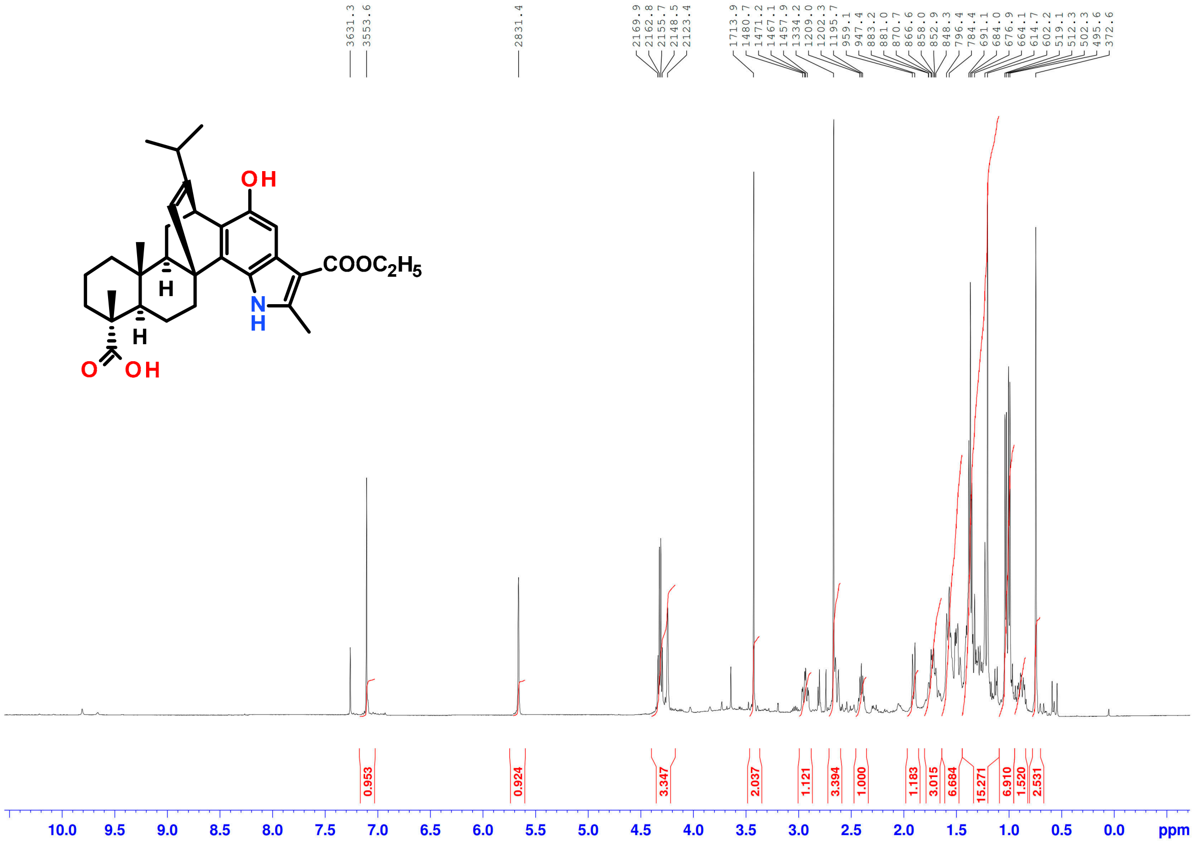

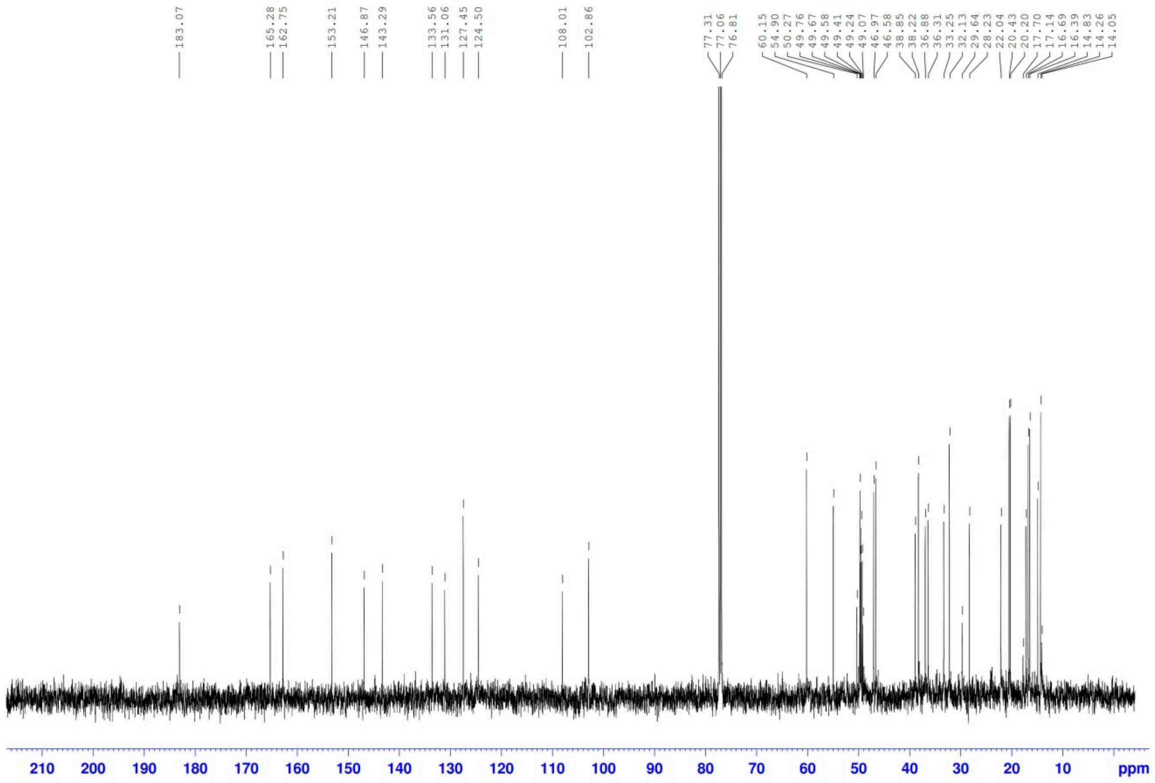

Figure S2. 2D – NMR spectra of compound **42** ($CDCl_3$).

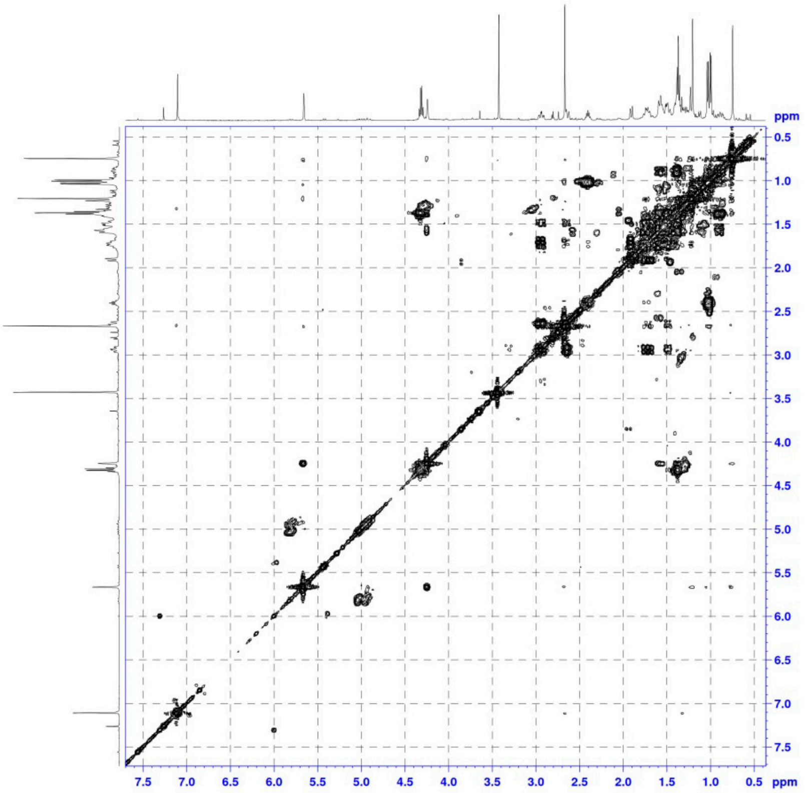

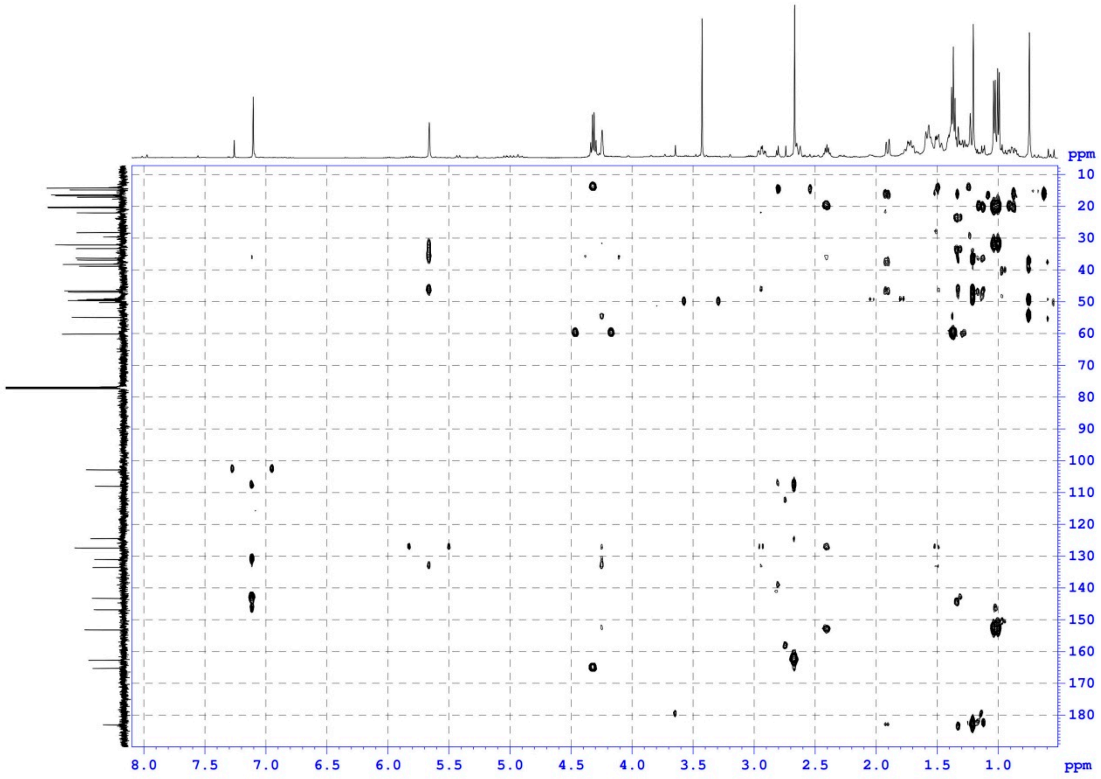

Figure S3. 2D – NMR spectra of compound **42** ($CDCl_3$).

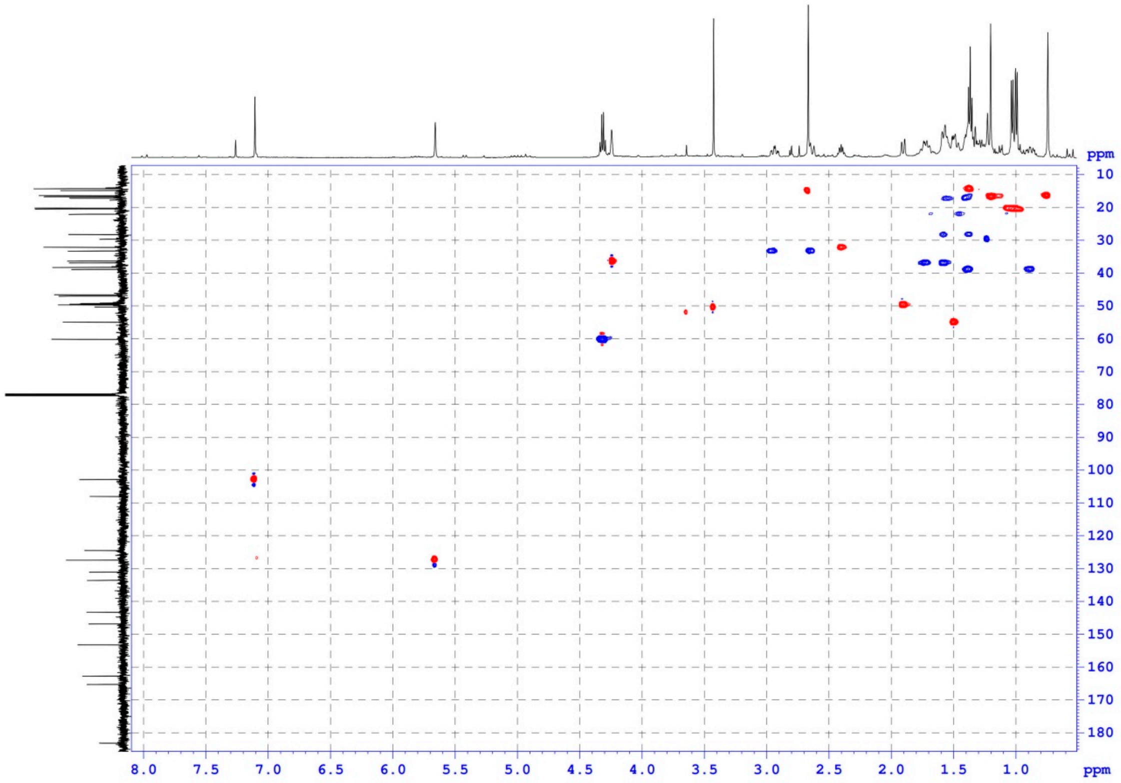

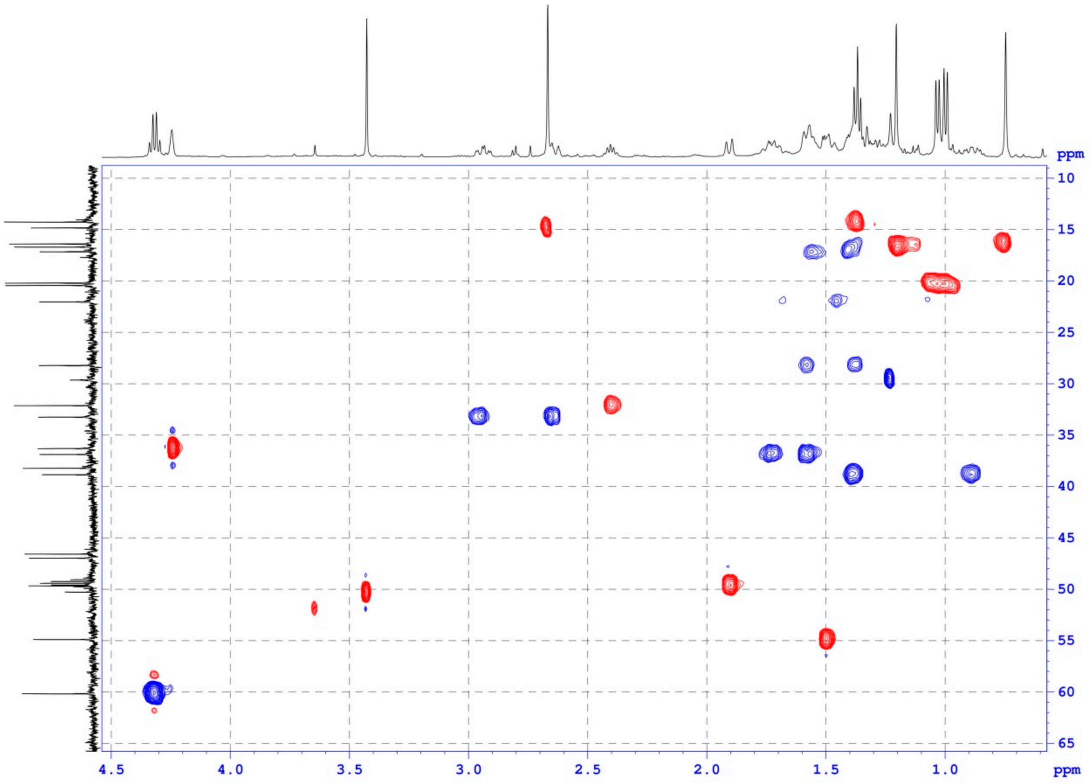

Figure S4. ^{1}H NMR and ^{13}C NMR spectra of compound **43** ($CDCl_3$).

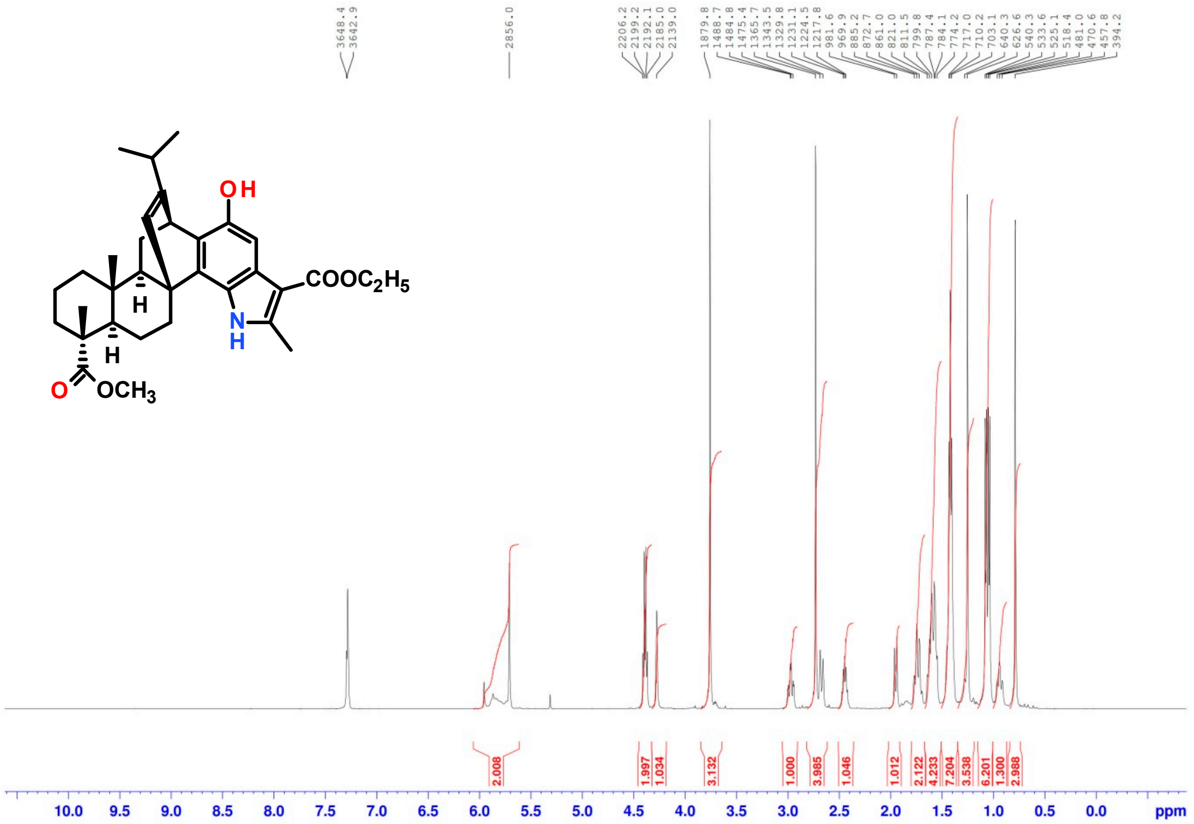

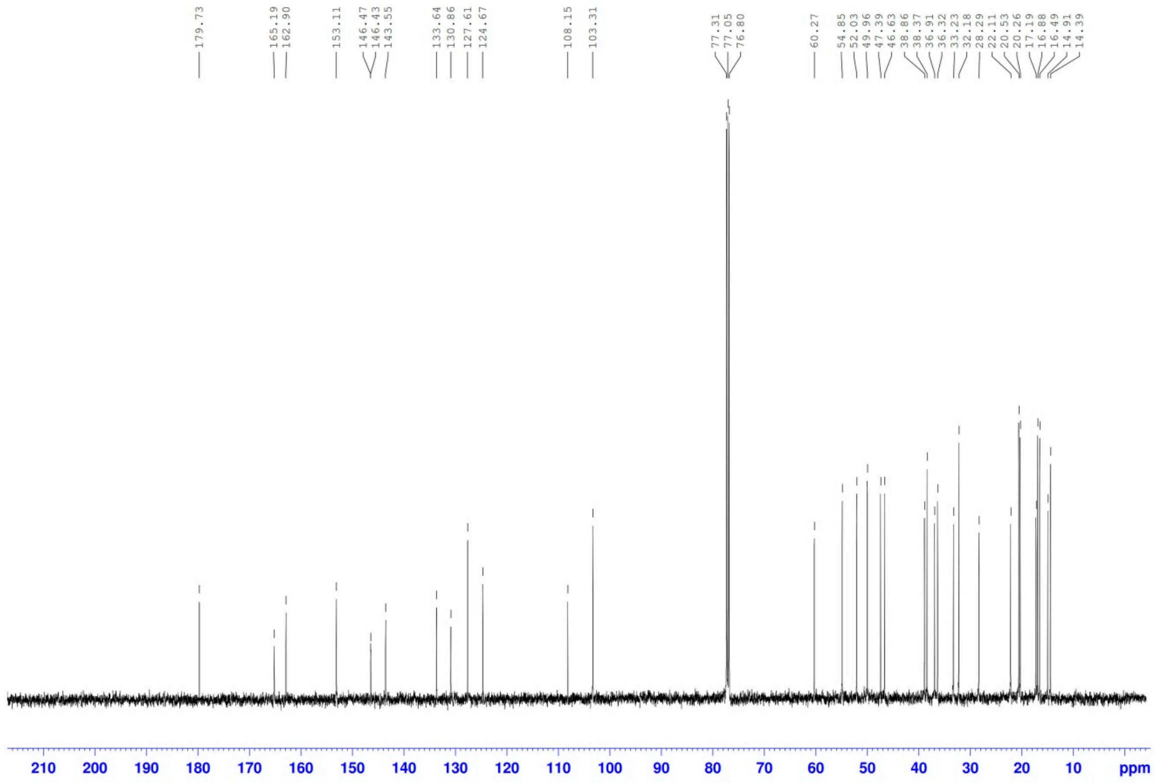

Figure S5. 2D – NMR spectra of compound **43** ($CDCl_3$).

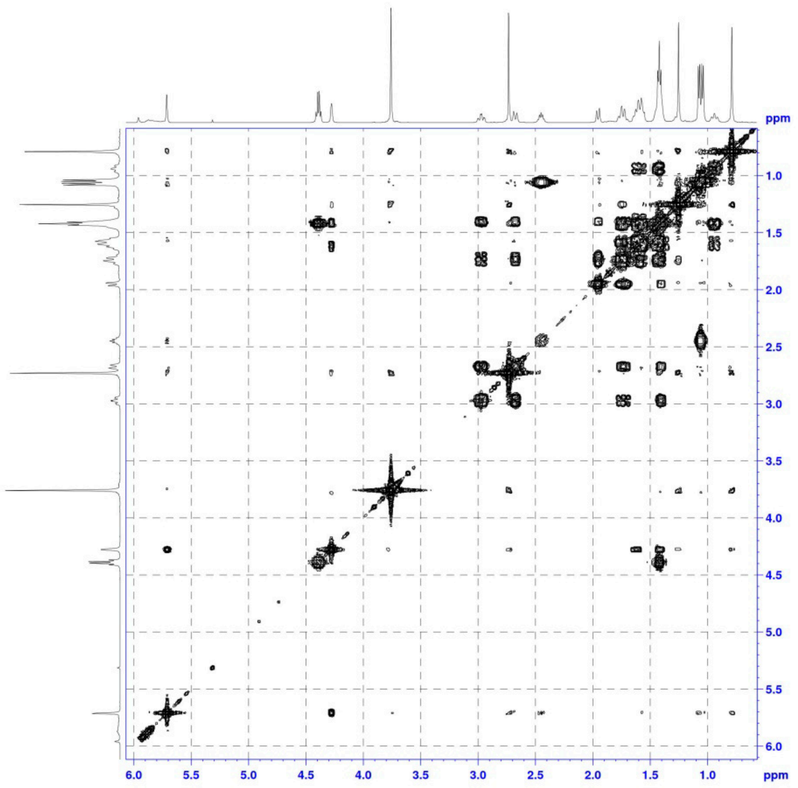

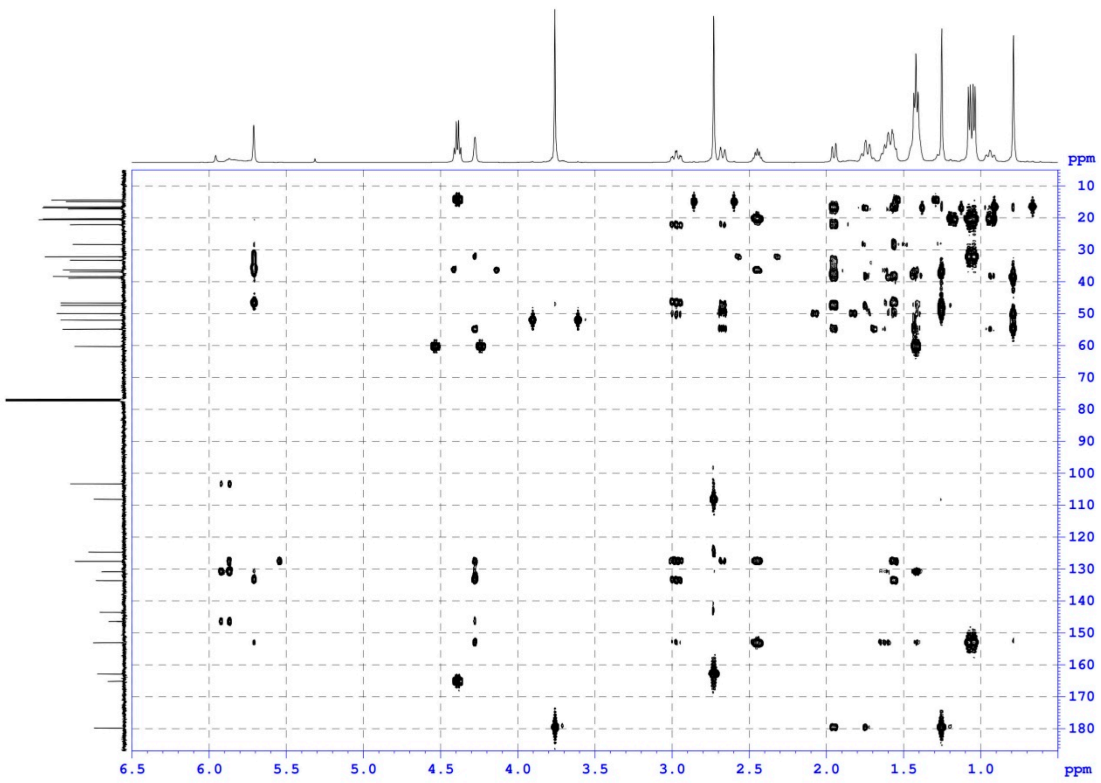

Figure S6. 2D – NMR spectra of compound **43** ($CDCl_3$).

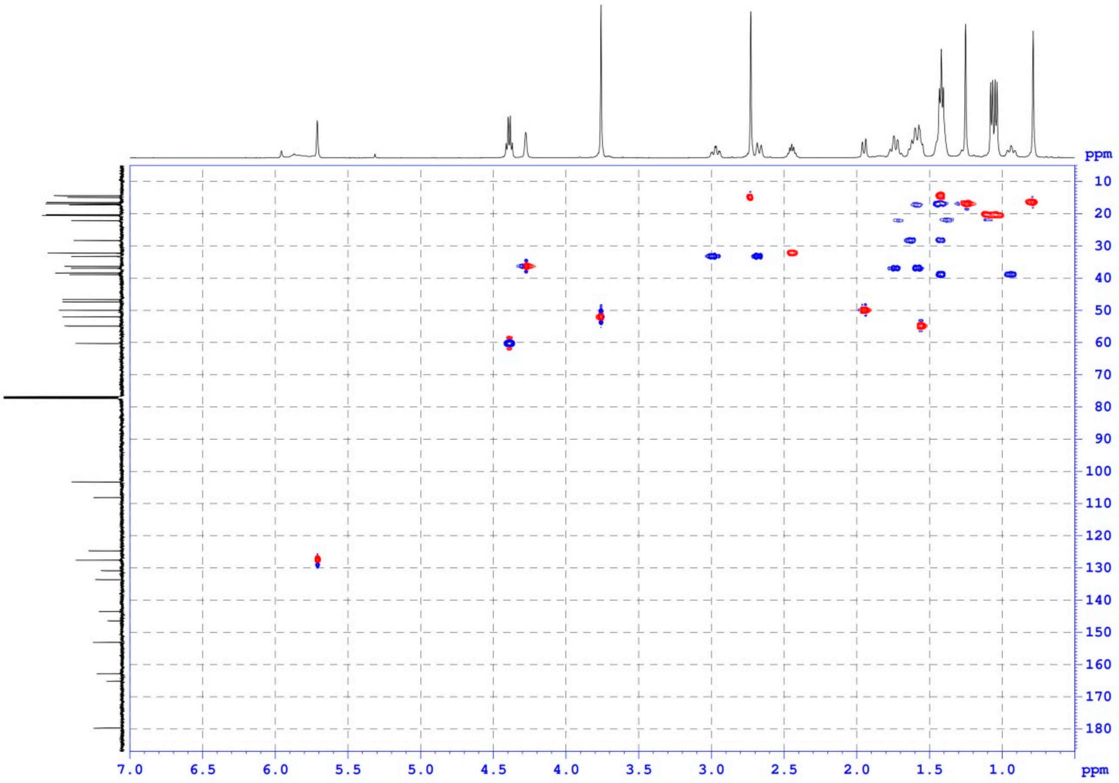

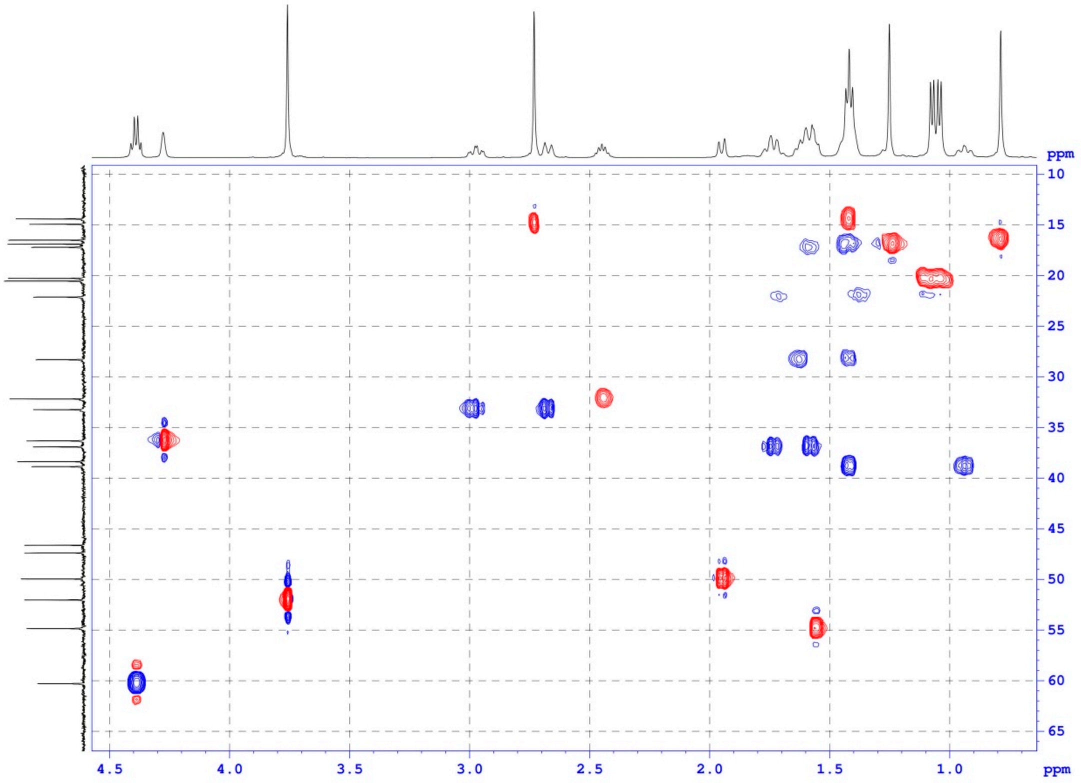

Figure S7. ^{1}H NMR and ^{13}C NMR spectra of compound **44** ($CDCl_3$ + MeOD).

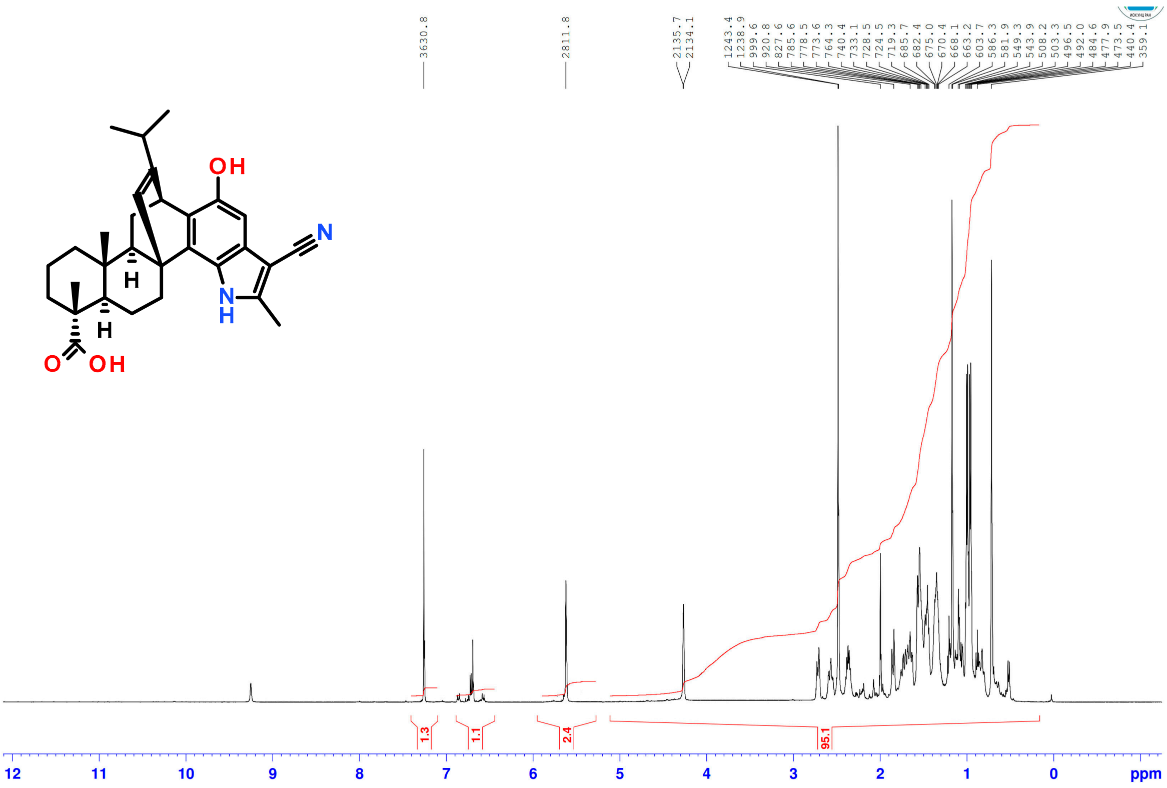

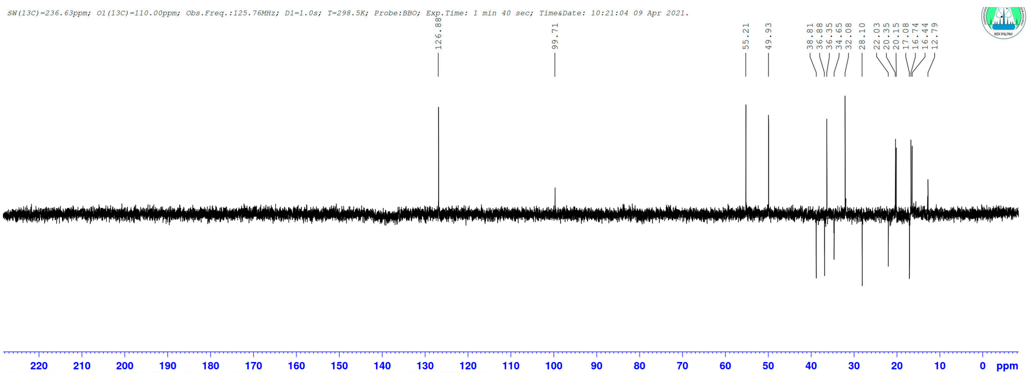

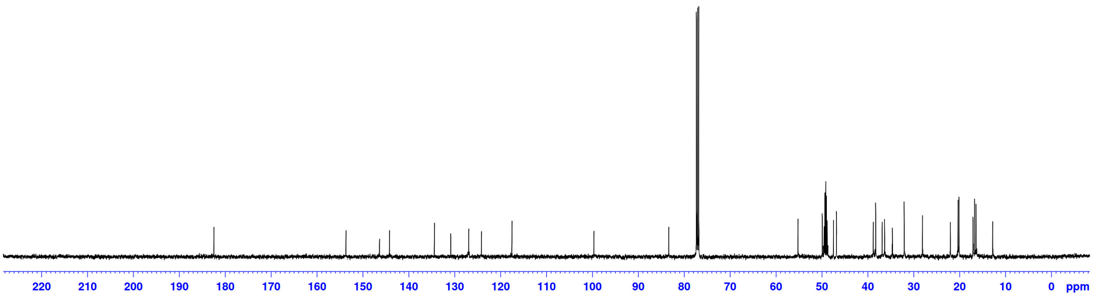

Figure S8. ^{1}H NMR and ^{13}C NMR spectra of compound **45** ($CDCl_3$).

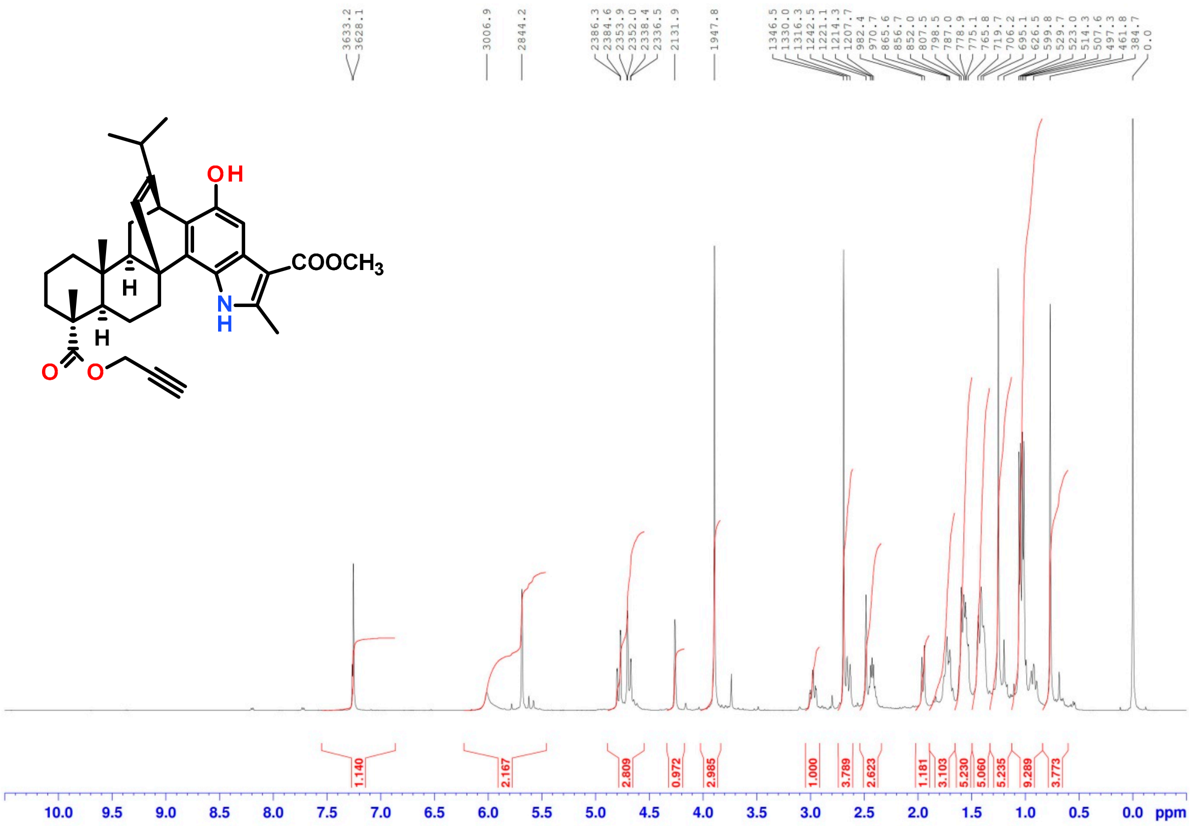

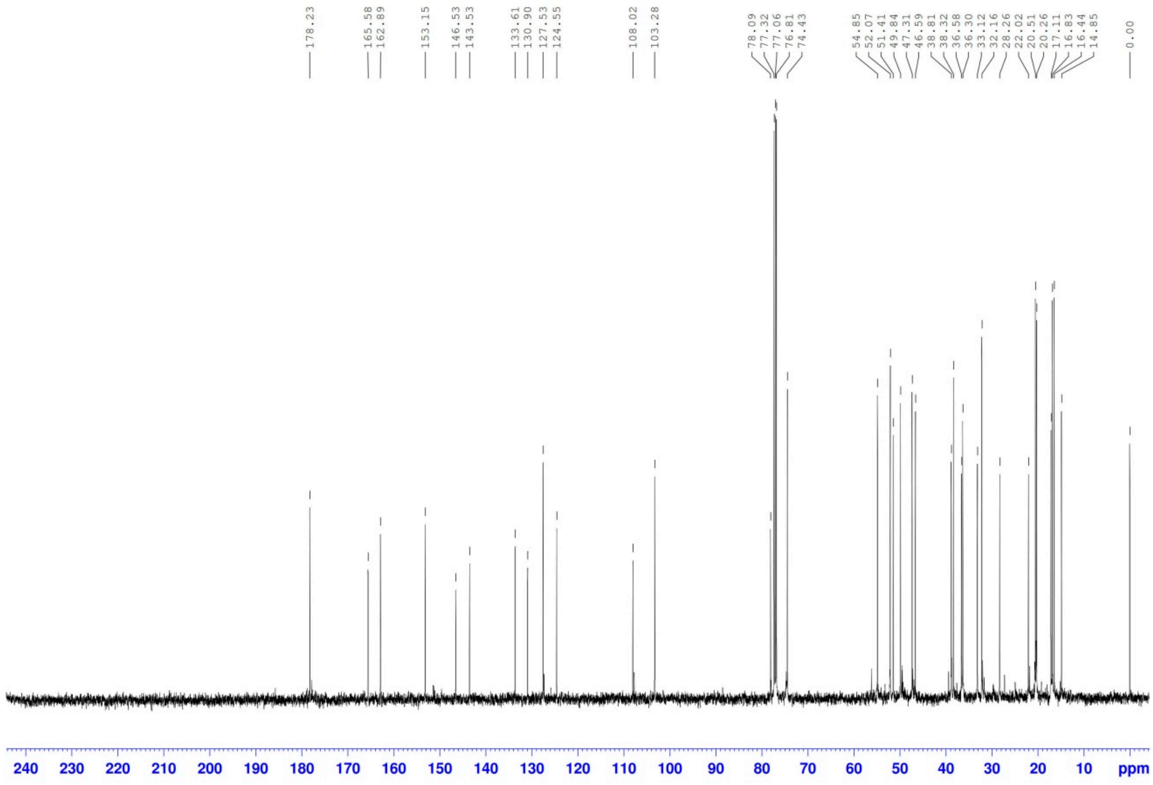

Figure S9. 2D – NMR spectra of compound **45** ($CDCl_3$).

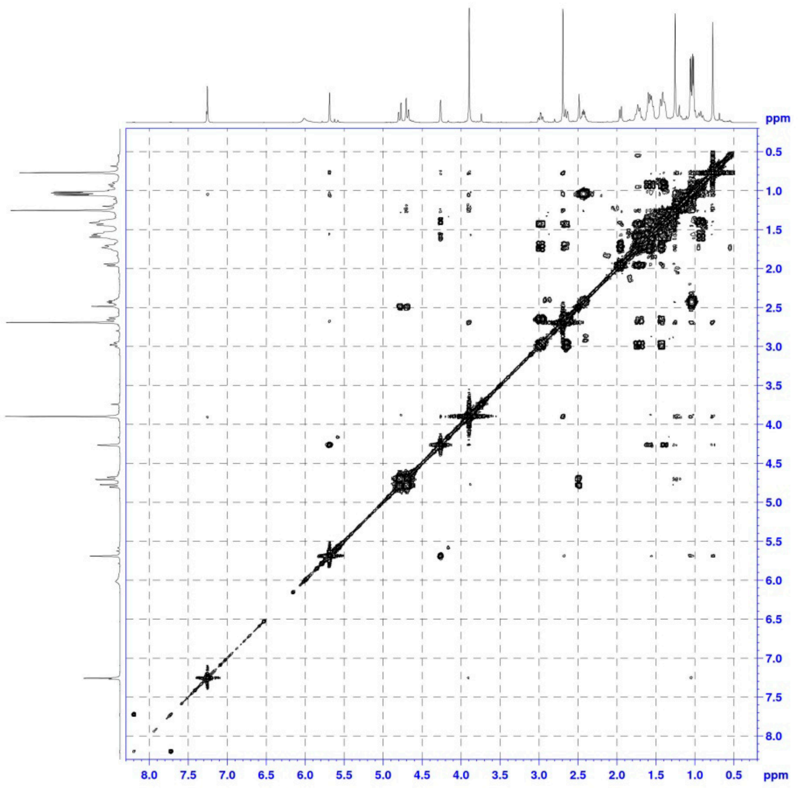

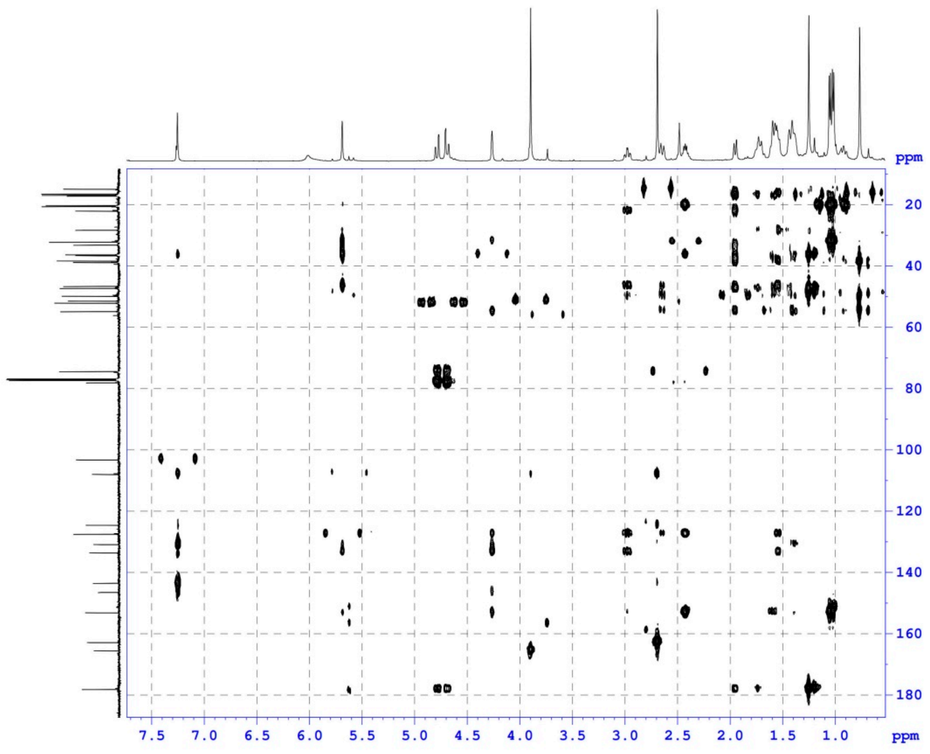

Figure S10. 2D – NMR spectra of compound **45** ($CDCl_3$).

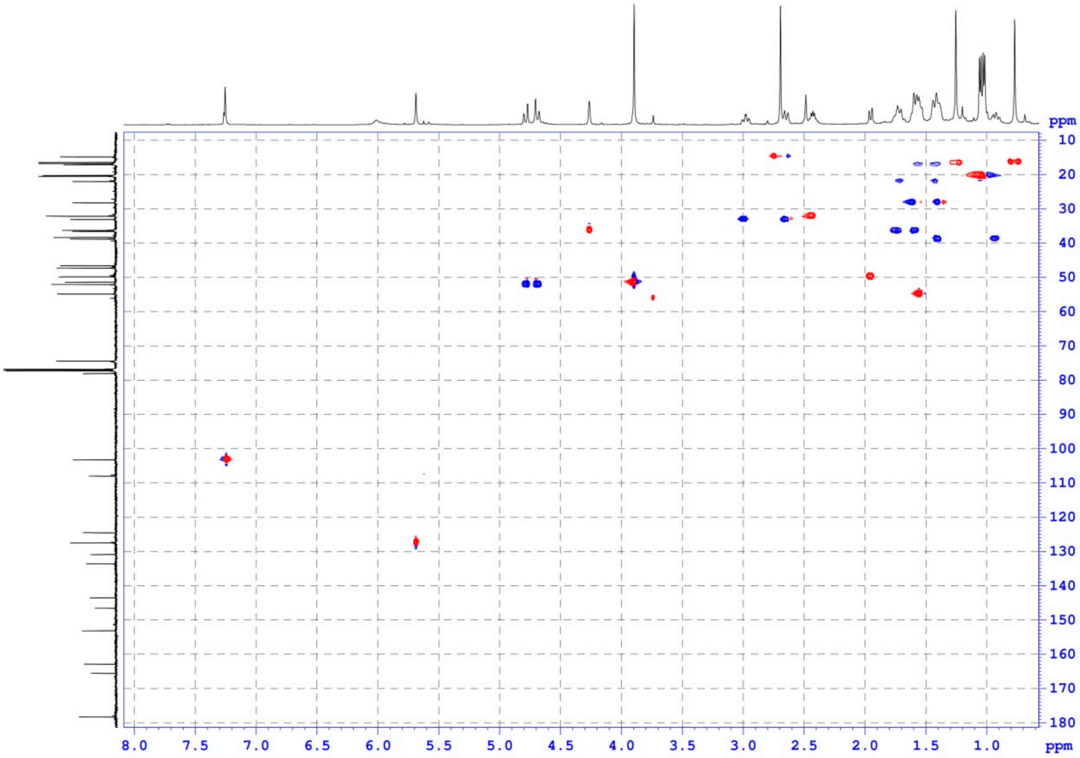

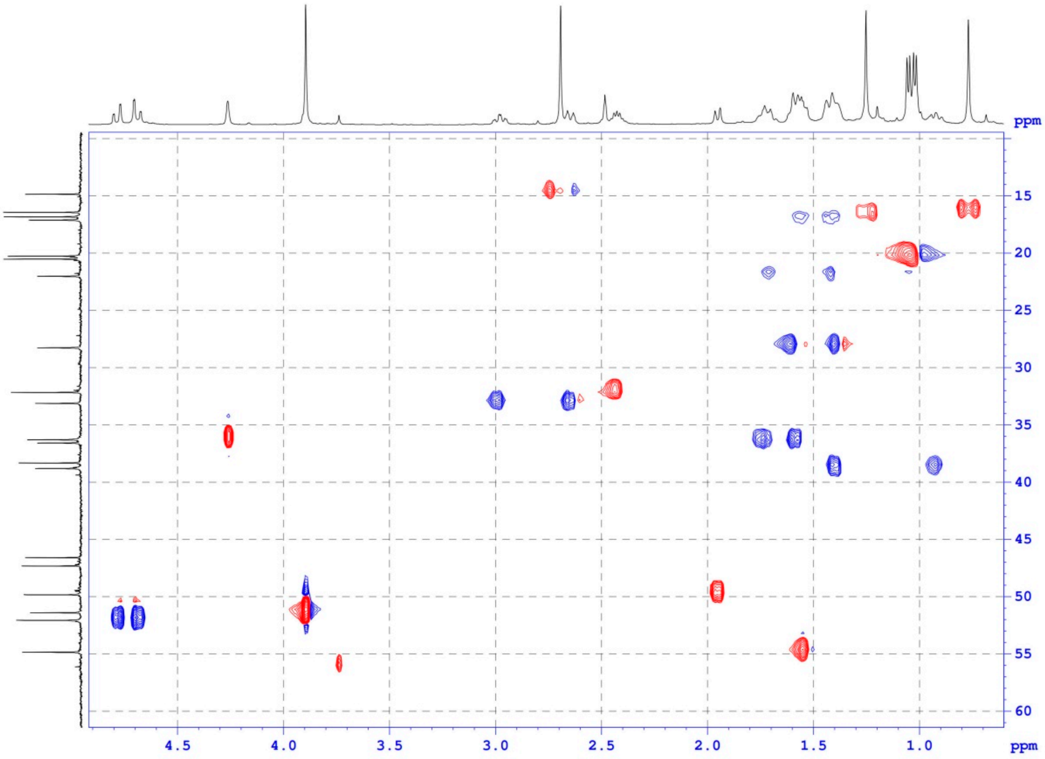

Figure S11. ^{1}H NMR and ^{13}C NMR spectra of compound **46** ($CDCl_3$).

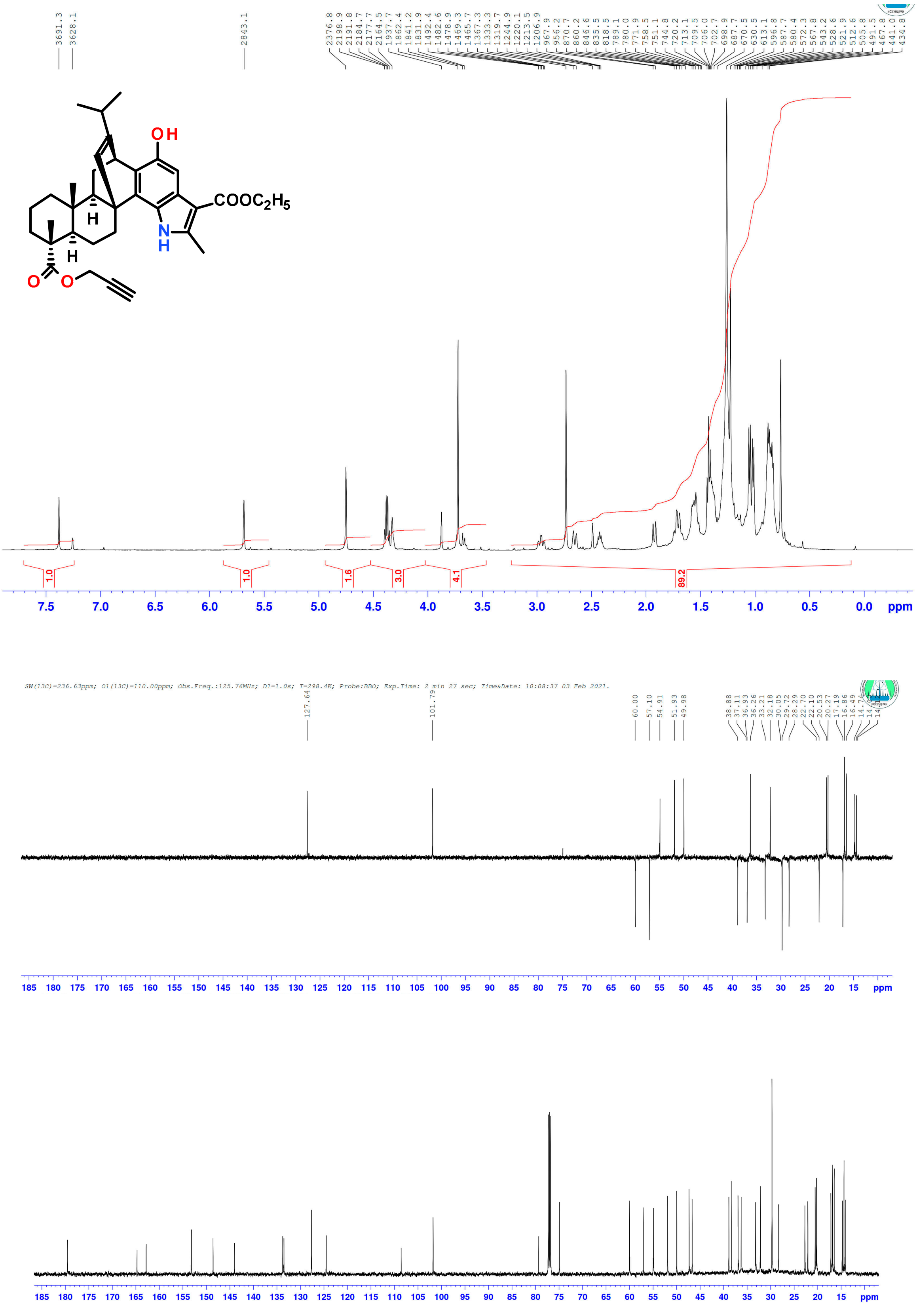

Figure S12. ^{1}H NMR and ^{13}C NMR spectra of compound **47** ($CDCl_3$).

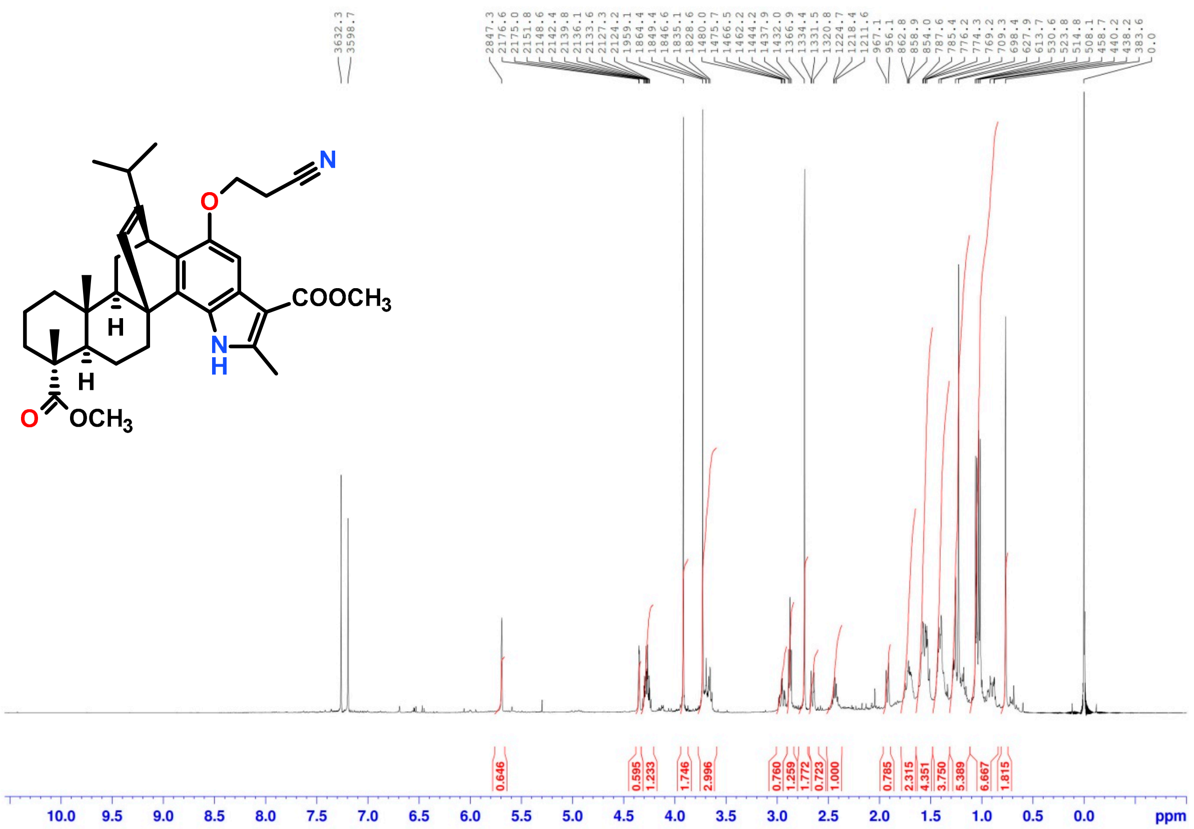

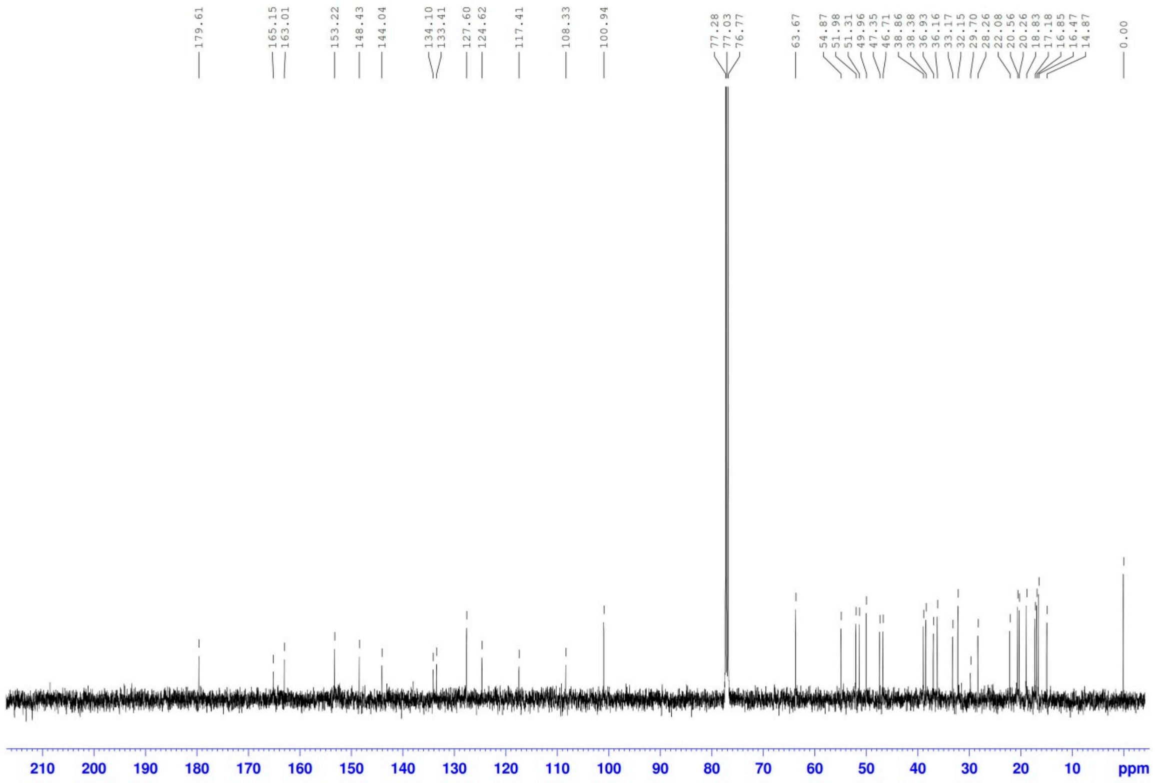

Figure S13. ^{1}H NMR and ^{13}C NMR spectra of compound **48** ($CDCl_3$).

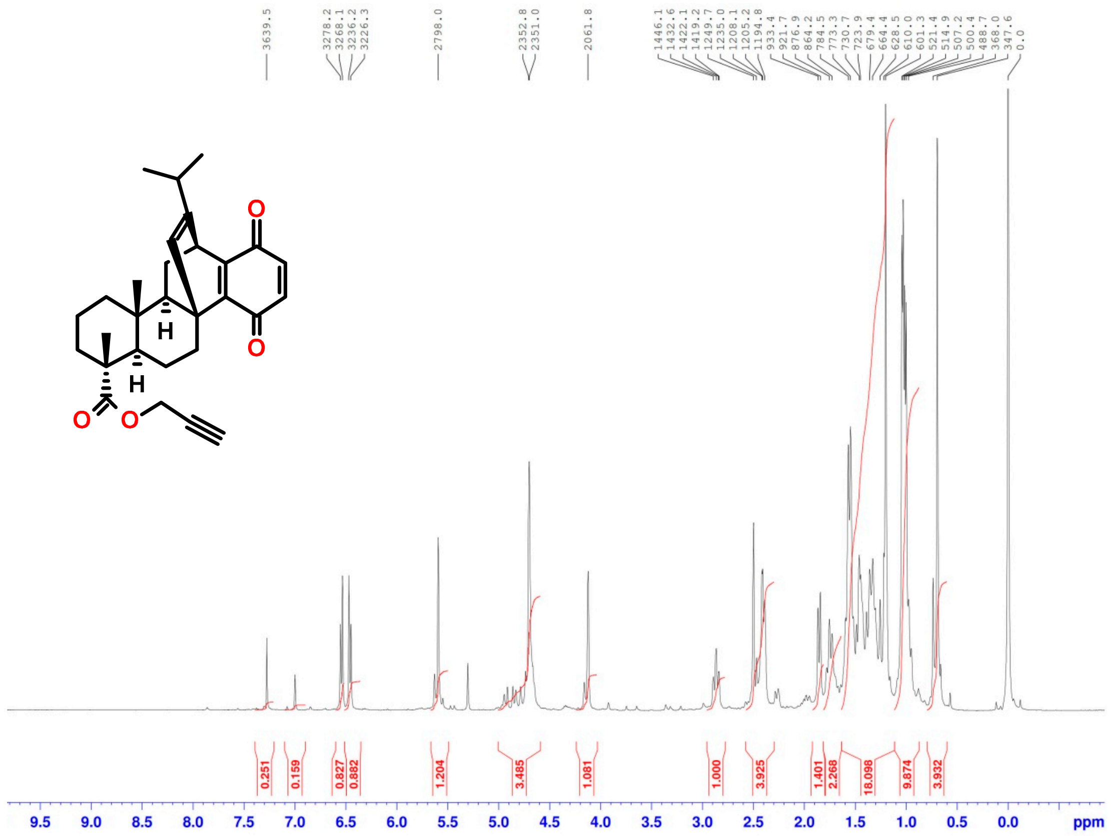

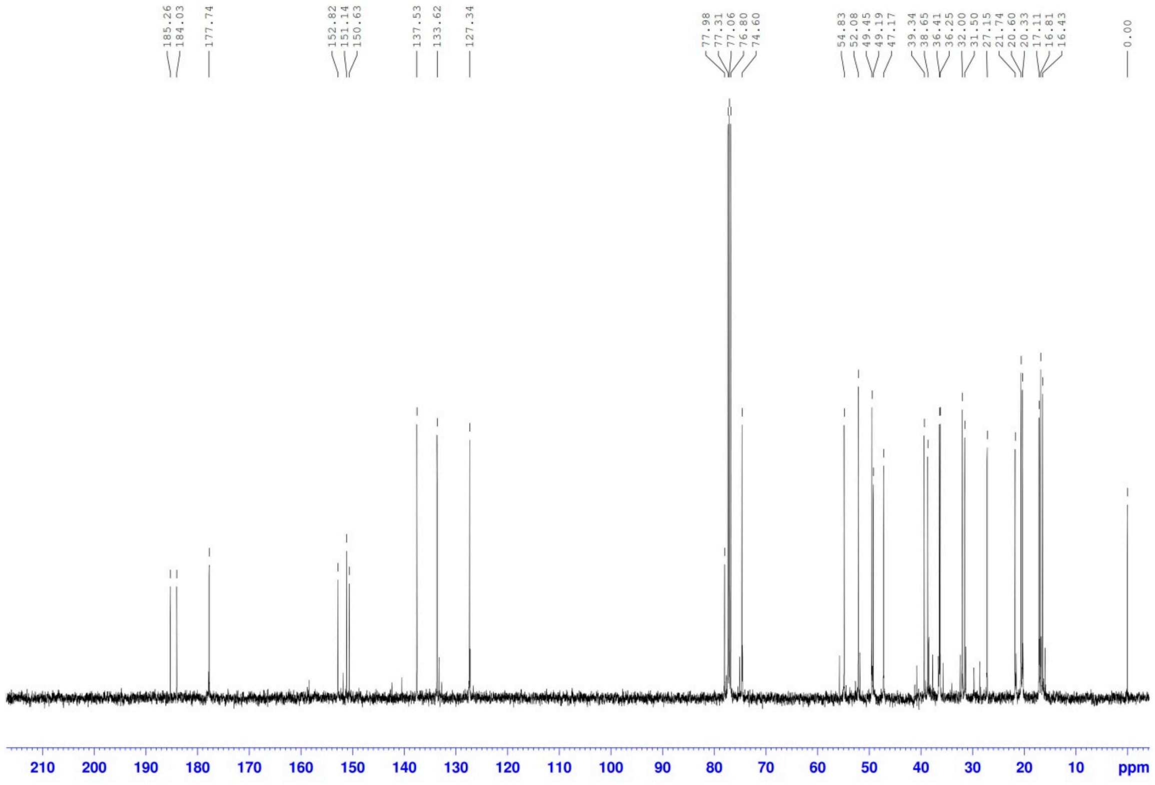

Figure S14. 2D – NMR spectra of compound **48** ($CDCl_3$).

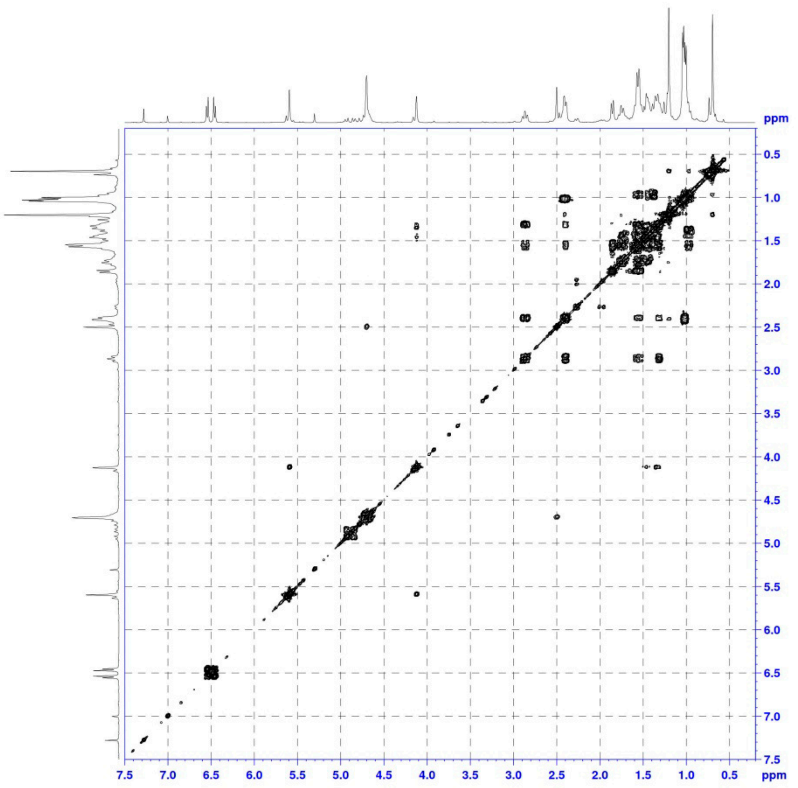

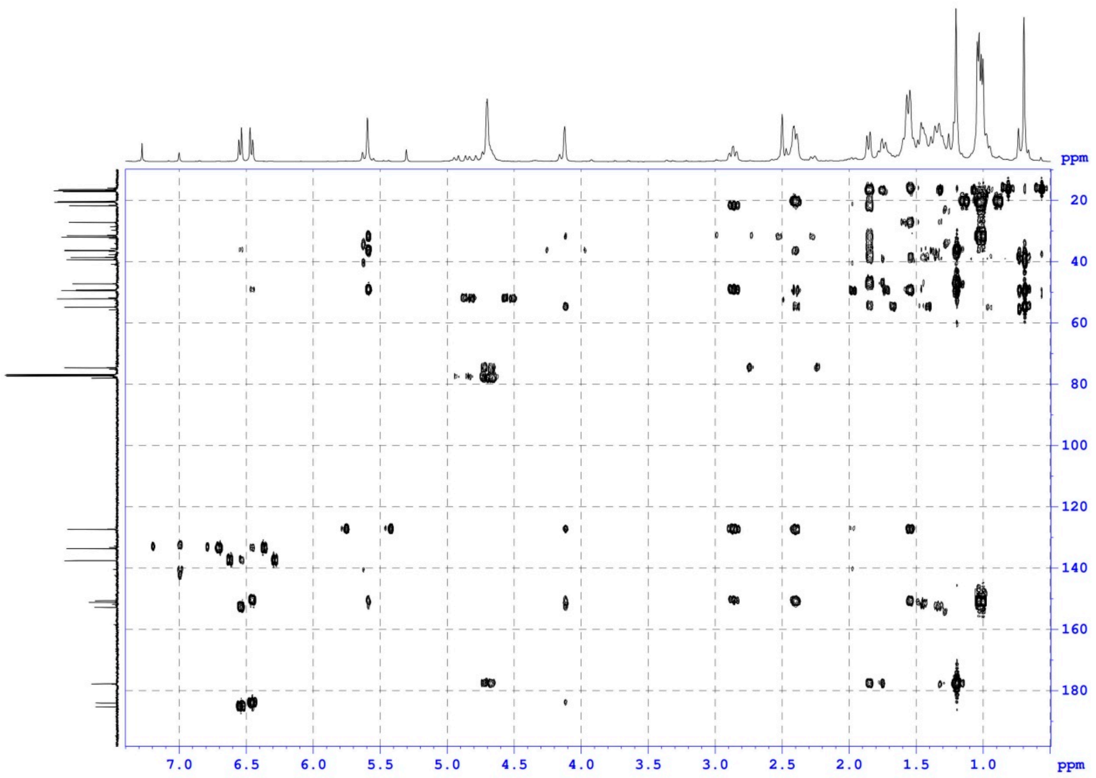

Figure S15. 2D – NMR spectra of compound **48** ($CDCl_3$).

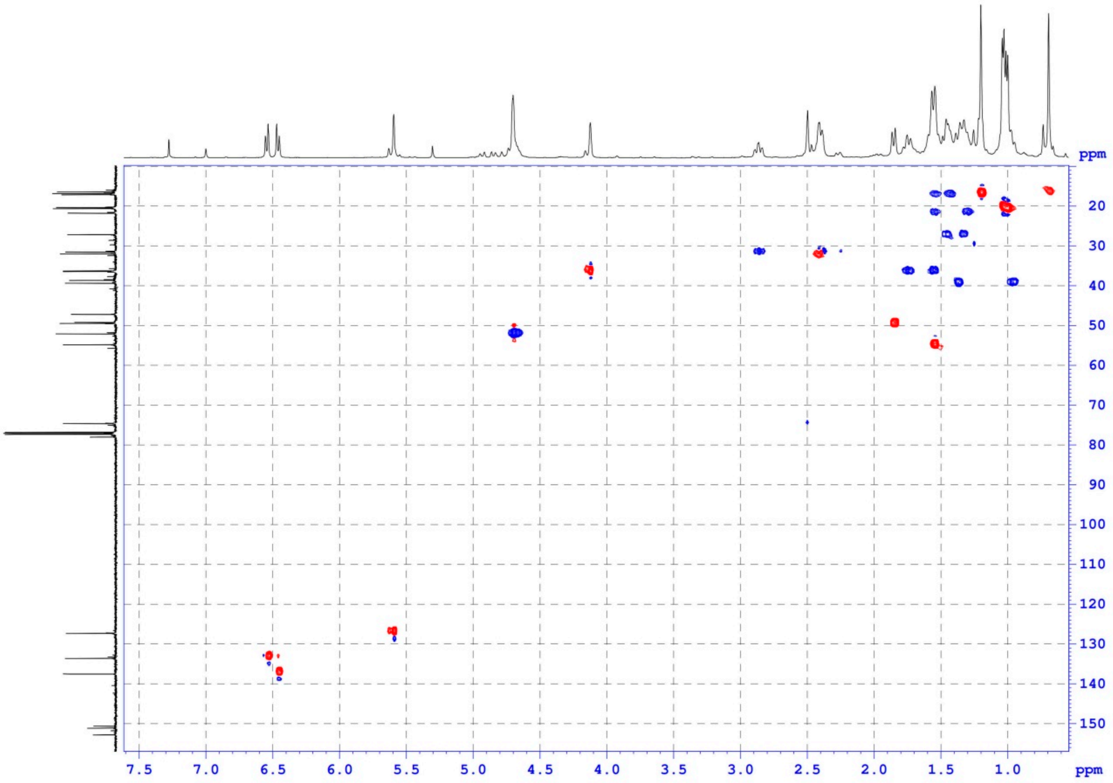

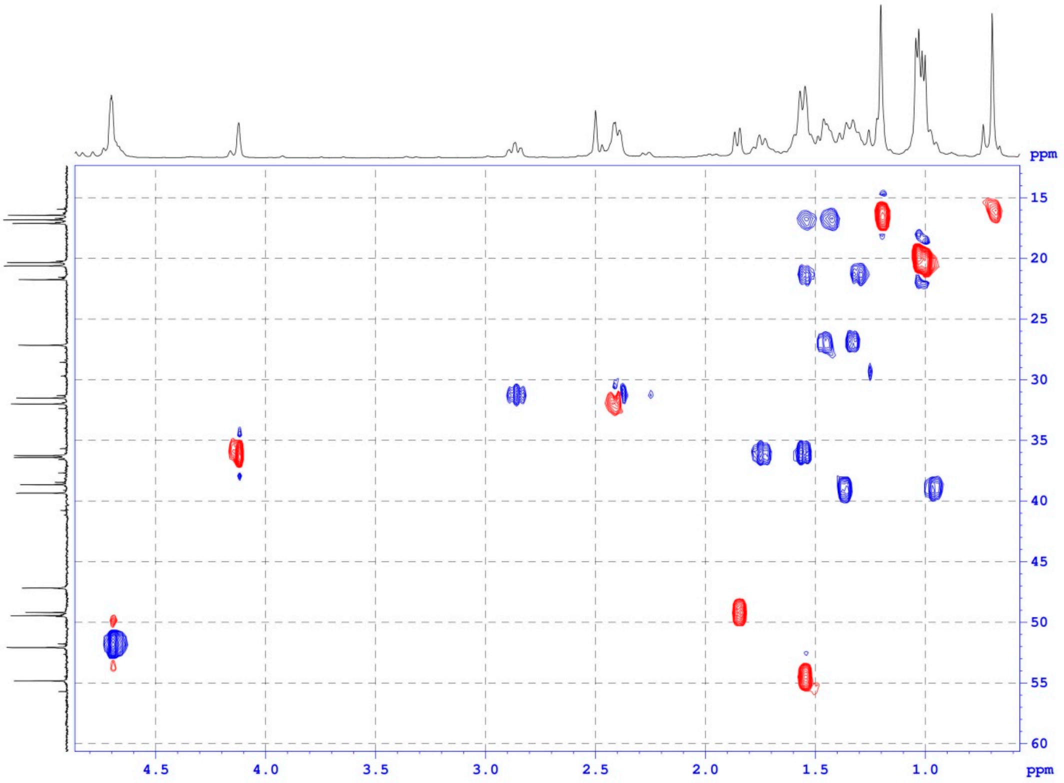

Figure S16. ^{1}H NMR and ^{13}C NMR spectra of compound **50** ($CDCl_3$).

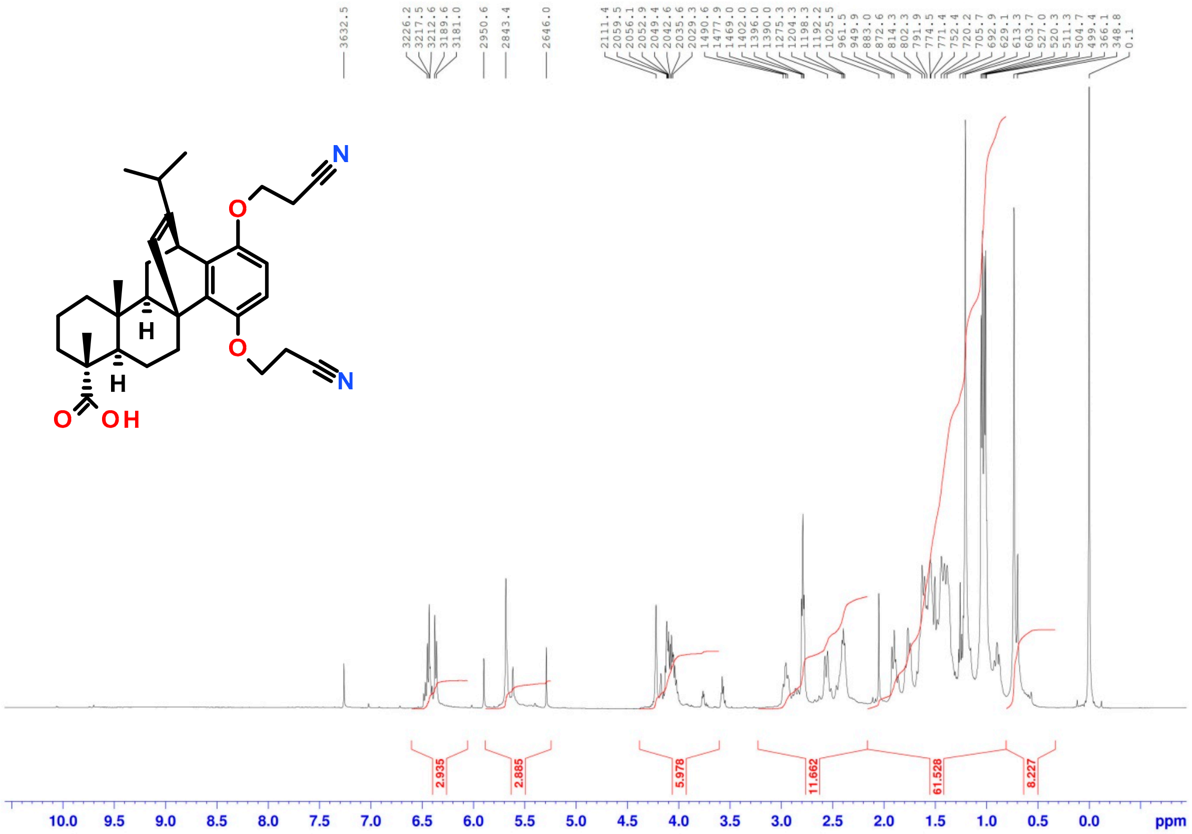

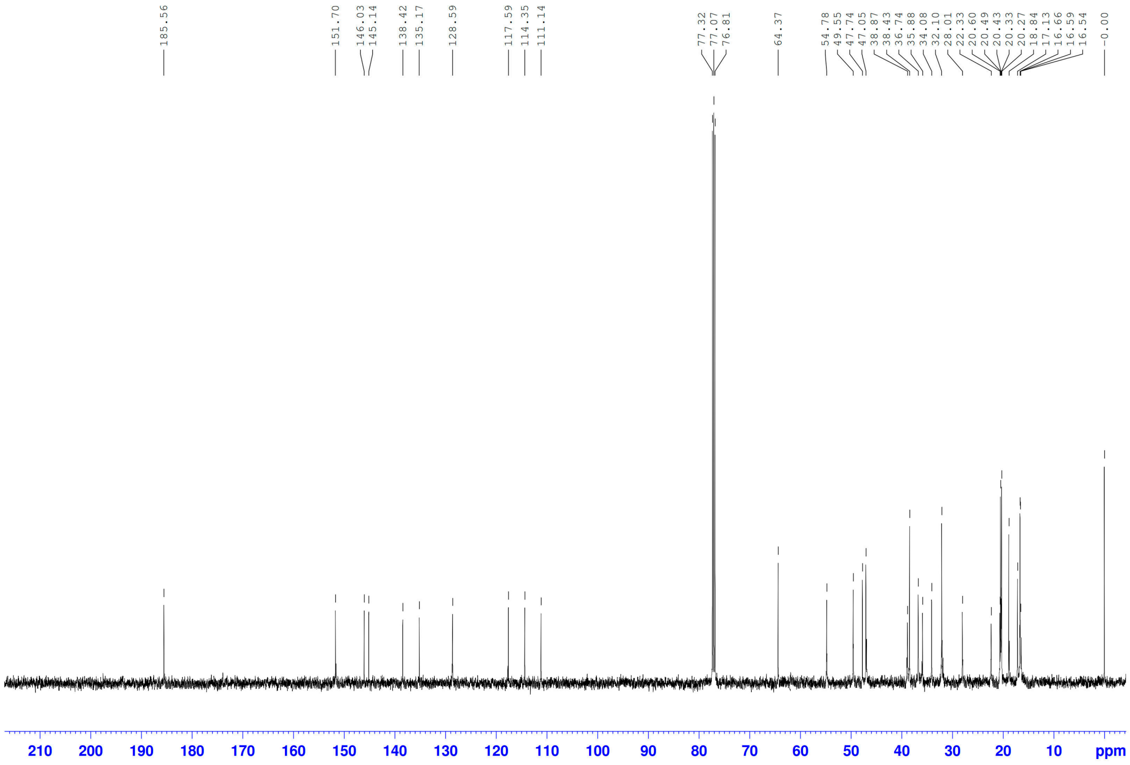

Figure S17. 2D – NMR spectra of compound **50** ($CDCl_3$).

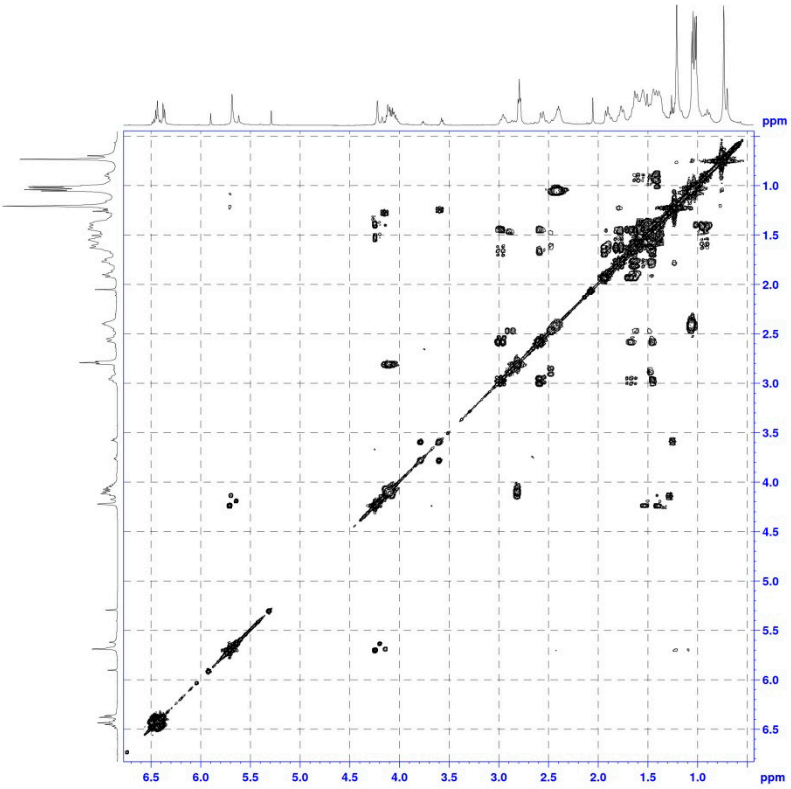

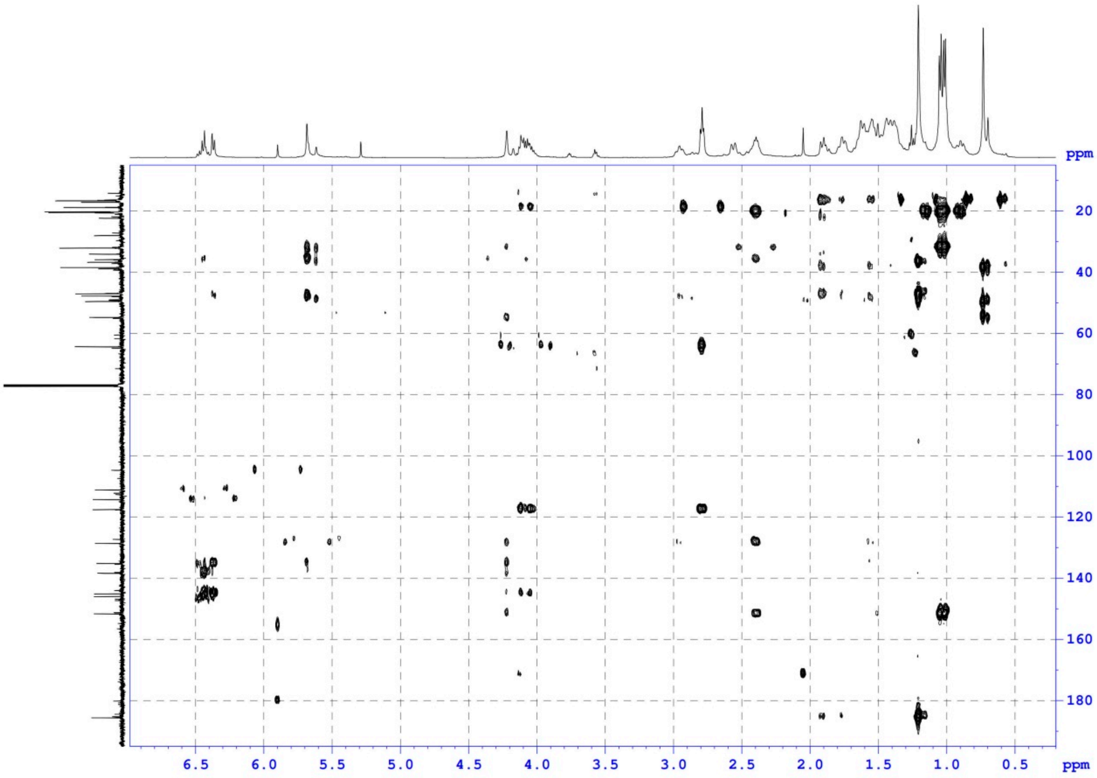

Figure S18. 2D – NMR spectra of compound **50** ($CDCl_3$).

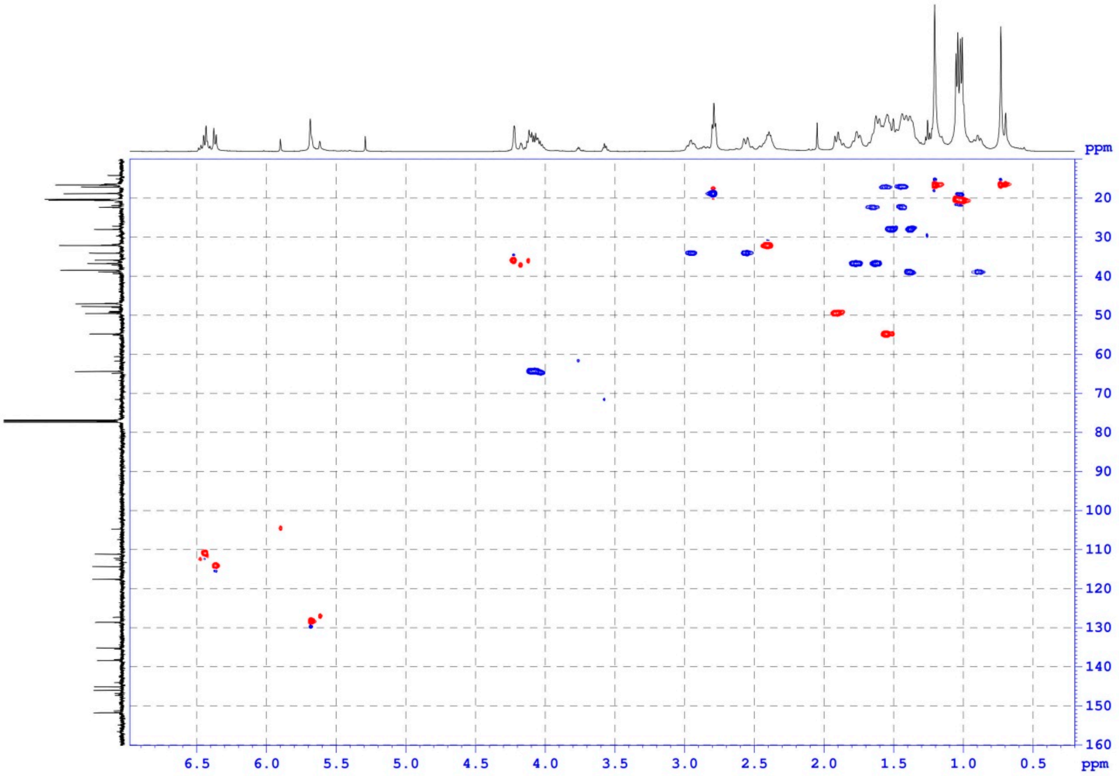

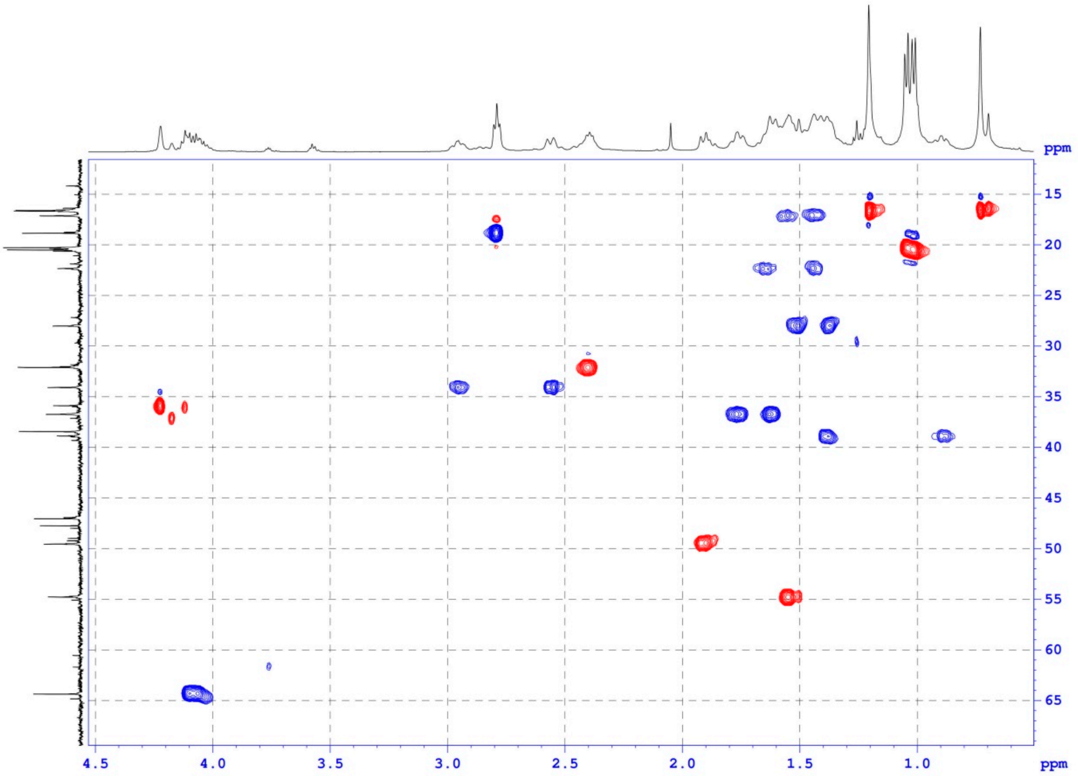

References

1. Nguyen Thi Thu Ha, Pham Van Cuong, Nguyen Thanh Tra, Le Thi Tu Anh, Ba Thi Cham, Ninh The Son, Chemical constituents from methanolic extract of *Garcinia mackeaniana* leaves and their antioxydant activity. Vietnam Journal of Science and Technology 58 (4) (2020) 411-418

2. Nguyen Thi Thu Ha, Pham Van Cuong, Le Thi Tu Anh, Nguyen Thanh Tra, Ba Thi Cham, Ninh The Son, Antimicrobacterial xanthones from *Garcinia mackeaniana leaves*. Vietnam J. Chem., 2020, 58(3), 343-348 DOI: 10.1002/vjch.2019000183

3. Mosmann T. Rapid colorimetric assay for cellular growth and survival: application to proliferation and cytotoxicity assays. J. Immunol Methods. 1983; 65:55-63. https://doi.org/10.1016/0022-1759(83)90303-4.

4. Kim Y.M., Wang M.H., Rhee H.I., A novel α-glucosidase inhibitor from pine bark. Carbohydr Res. 339 (2004) 715–717. 10.1016/j.carres.2003.11.005.

5. Li T., Zhang X.D., Song Y.W., Liu J.W., A microplate-based screening method for α-glucosidase inhibitors, Nat. Prod. Res. Dev. 10 (2005) 1128–1134.

6. MarvinSketch 18.8.0, ChemAxon Ltd., 2018.

7. Spasov A.A., Babkov D.A., Osipov D.V., Klochkov V.G., Prilepskaya D.R., Demidov M.R., Osyanin V.A., Klimochkin Y.N., Synthesis, in vitro and in vivo evaluation of 2-aryl-4H-chromene and 3-aryl-1H-benzo[f]chromene derivatives as novel α-glucosidase inhibitors, Bioorg Med Chem Lett. 29 (2019) 119–123. https://doi.org/10.1016/j.bmcl.2018.10.018.

8. Trott O., Olson A.J., AutoDock Vina: Improving the speed and accuracy of docking with a new scoring function, efficient optimization, and multithreading, Journal of Computational Chemistry. 31 (2009) 455–461. https://doi.org/10.1002/jcc.21334.

9. Discovery Studio Visualizer 17.2.0.16349, Dassault Systemes Biovia Corp., 2016.
